# Multi-marker analysis of *Fasciola gigantica* from cattle and buffalo across Pakistan reveals high levels of genetic diversity and novel haplotypes

**DOI:** 10.1017/S0031182025100693

**Published:** 2025-09

**Authors:** Maria Komal, Kiran Afshan, Sabika Firasat, Jane E. Hodgkinson, Krystyna Cwiklinski

**Affiliations:** 1 Department of Zoology, Faculty of Biological Sciences, Quaid-i-Azam University, Islamabad, Pakistan; 2 Institute of Infection, Veterinary and Ecological Sciences, University of Liverpool, Liverpool, UK

**Keywords:** *Fabp*, *Fasciola gigantica*, genetic diversity, mitochondrial NADH dehydrogenase, molecular markers, *Pepck*, RAPD

## Abstract

Molecular analyses of geographically dispersed *Fasciola* spp. isolates based on ribosomal, mitochondrial and nuclear molecular markers have revealed high levels of genetic diversity within liver fluke populations. To investigate the *Fasciola* population substructure across Pakistan 4 molecular markers were compared (fatty acid binding protein, *fabp;* phosphoenolpyruvate carboxykinase, *pepck*; random amplified polymorphic DNA, RAPD; mitochondrial NADH dehydrogenase, *mt-nd1*). Adult parasites (*n* = 595) were collected from buffalo and cattle across 4 provinces in Pakistan (Baluchistan, Gilgit Baltistan, Khyber Pakhtunkhwa, Punjab). Species classification of all 595 parasites was confirmed by the 3 gel-based markers (*pepck, fabp* and RAPD) as *F. gigantica*, except for the *fabp* marker which unexpectedly could not be amplified in 274 parasites (46%). Analysis of a subset of samples indicates the potential for mis-priming due to multiple genomic loci that match the *fabp* primer sequences resulting in negative PCR products in some cases. Sequence analysis of the *mt-nd1* PCR products identified 29 haplotypes within the samples from Pakistan, the majority of which are unique to this study. None of the 29 haplotype sequences were identified in samples from Africa, highlighting the genetic diversity between geographically disparate liver fluke populations. Inconsistencies between *Fasciola* spp. molecular markers in this study highlights the need for multiple markers, validated on large numbers of geographically disparate parasites, to generate robust analyses of liver fluke genetic diversity. This study echoes other *Fasciola* spp. population studies and highlights the genetic diversity of *F. gigantica* populations in Pakistan that is comparable to observations of diversity throughout Asia.

## Graphical abstract

**Figure:**
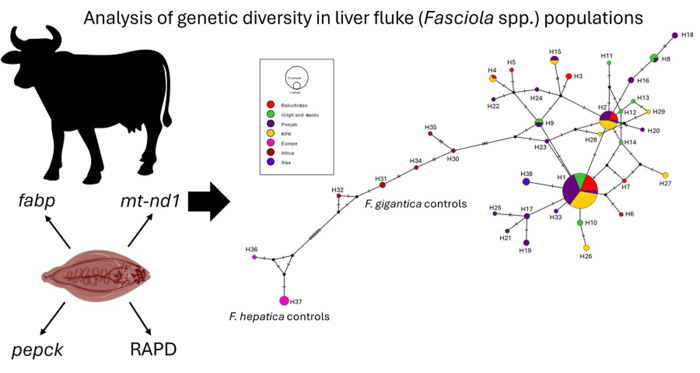

## Introduction

The liver fluke parasites, *Fasciola hepatica* and *Fasciola gigantica*, are the causative agents of fasciolosis, an economically important disease of people and their livestock worldwide. Liver fluke infection and the ensuing disease are estimated to cost US $3.2 billion a year worldwide, driven by the negative impact on animal growth, wool and milk production and fertility, and the costs associated with parasite control (Mas-Coma et al, 2005; MacGillivray et al, 2013; Mazeri et al, 2017; Nyirenda et al, 2019; Lalor et al, 2021; Utrera-Quintana et al, 2022). Control is reliant on the use of anthelmintic drugs, predominately triclabendazole. However, widespread resistance to this effective anthelmintic is limiting its use (Alvarez et al, 2021), requiring controlled and appropriate use of triclabendazole on farm and development of novel control strategies.

*Fasciola* spp. are typically restricted geographically, with *F. hepatica* distributed in more temperate areas and *F. gigantica* favouring tropical climates; however, there are areas where the two species co-exist, such as Pakistan (Afshan et al, 2014b), resulting in the potential for species hybridization. Coupled with climate change and anthropogenic pressures (Afshan et al, 2014a), this has implications for the severity of disease, and potential spread of genetic loci associated with drug resistance within liver fluke populations. Parasite prevalence studies therefore play an important role in determining which *Fasciola* spp. are circulating and monitoring the potential spread of infection/disease.

While there are morphological differences between *F. hepatica* and *F. gigantica*, high levels of morphometric variability amongst parasites within the same species limits the reliability of this method for liver fluke species identification. Molecular markers based on ribosomal, mitochondrial and nuclear regions of the liver fluke genomes are more commonly used for both species identification and analysis of genetic diversity within liver fluke populations. Differentiation of *Fasciola* spp. can be visually determined by gel electrophoresis for the markers based on random amplified polymorphic DNA (RAPD; McGarry et al, 2007), phosphoenolpyruvate carboxykinase (*pepck*; Shoriki et al, 2016), and the recently reported fatty acid binding protein (*fabp*; Okamoto et al, 2022). The ribosomal and mitochondrial markers including internal transcribed spacer-2 (ITS-2; Adlard et al, 1993), NADH dehydrogenase (*mt-nd1*; Itagaki et al, 1998) and cytochrome C oxidase subunit 1 (*mt-cox1*; Itagaki et al, 1998), require sequencing, often using next-generation sequencing technologies, to facilitate differentiation of the *Fasciola* spp. However, this approach does facilitate more in-depth analysis of genetic diversity within liver fluke populations in addition to the differentiation of *Fasciola* spp. These markers have shown that liver fluke populations throughout the world display high levels of genetic diversity (Beesley et al, 2017; Aghayan et al, 2019; Vazquez et al, 2021; Hecker et al, 2025). This is also mirrored by the recent sequencing of the *Fasciola* spp. genomes that revealed these parasites have large genomes (approximately 1 Gb), that display high levels of gene polymorphism at the individual fluke level (Cwiklinski et al, 2015; Choi et al, 2020; Luo et al, 2021).

In this study, the genetic diversity/substructure of *Fasciola spp.* populations across Pakistan was assessed using 4 molecular markers, namely *pepck, fabp*, RAPD and *mt-nd1*, using DNA extracted from liver fluke parasites collected from cattle and buffalo across 4 provinces in Pakistan (Baluchistan, Gilgit Baltistan, Khyber Pakhtunkhwa, Punjab). All the parasites analysed were identified as *F. gigantica* by all 4 markers. Unexpectedly, our analysis using the new *Fasciola* spp. marker, *fabp*, revealed inconsistencies in the amplification of this marker, that warrant further investigation. Based on *mt-nd1* sequence analysis, the genetic sub-structure within these liver fluke populations was investigated and compared to previous studies carried out in Pakistan. This analysis identified several novel haplotype sequences, confirming the high levels of genetic diversity within *F. gigantica* populations in Pakistan.

## Materials and methods

### Study area and sampling

**Figure 1.**
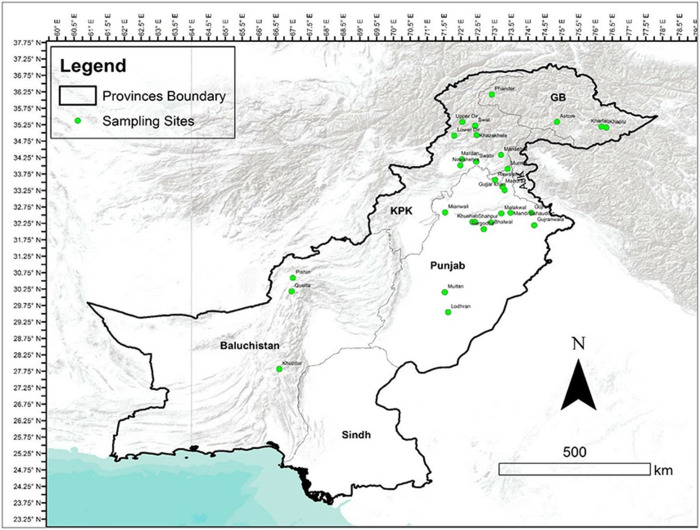
Map of study area across the 4 provinces in Pakistan. The sampling sites are highlighted in green.

A total of 595 adult liver fluke samples were collected from cattle (203) and buffalo (392) across the 4 main provinces in Pakistan, namely Baluchistan, Gilgit Baltistan, Khyber Pakhtunkhwa (KPK) and Punjab (Figure 1; Table 1). Within each province, adult liver flukes were recovered from several districts as follows: (a) Baluchistan–Khuzdar, Pishin, Quetta and Khuzdar districts: seven fluke per liver were recovered from 16 infected buffalo and 2 infected cattle; (b) Gilgit Baltistan–Astore, Khaplu, Kharfaq and Phander districts: 4 fluke per liver recovered from 11 infected buffalo and two infected cattle; (c) KPK–Lower Dir, Upper Dir, Manshera, Nowshehra, Sawabi and Swat districts: 5 fluke per liver were recovered from 15 infected buffalo and 18 infected cattle; (d) Punjab–Bhalwal, Gijjar Khan, Gujranwala, Lodhran, Mianwali, Multan, Rwalpindi, Sargodha and Shahpur districts: 7 fluke per liver were recovered from 23 infected buffalo and 13 infected cattle. The adult flukes were washed in phosphate-buffered saline (PBS) and preserved in 70% ethanol for molecular analysis.

**Table 1.** Sampling sites across four provinces in Pakistan (Baluchistan, Gilgit Baltistan, Khyber Pakhtunkhwa (KPK) and Punjab)

Province	Area	Latitude	Longitude	Elevation (m)
**Baluchistan**	Khuzdar	27.8165° N	66.6057° E	1,237
	Pishin	30.5897° N	67.0107° E	1,555
	Quetta	30.1798° N	66.9750° E	1,680
**Gilgit Baltistan**	Astore	35.3570° N	74.8624° E	2,546
	Khaplu	35.1611° N	76.3319° E	2,600
	Kharfaq	35.190906°N	76.181643°E	4,600
	Phander	36°10′10″N	72°56′02″E	2,904
**KPK**	Lower Dir	34.9161° N	71.8097° E	1,112
	Upper Dir	35.3356° N	72.0468° E	1,420
	Manshera	34.3313° N	73.1980° E	1,088
	Nowshehra	34.0105° N	71.9876° E	552
	Sawabi	34.1241° N	72.4613° E	340
	Swat	35.2227° N	72.4258° E	3,220
**Punjab**	Bhalwal	32.2751° N	72.9047° E	196
	Gujjar Khan	33.251299° N	73.30602° E	461
	Gujranwala	32.1877° N	74.1945° E	231
	Lodhran	29.5467° N	71.6276° E	117
	Mianwali	32.5839° N	71.5370° E	210
	Multan	30.1575° N	71.5249° E	122
	Rawalpindi	33.5651° N	73.0169° E	508
	Sargodha	32.0740° N	72.6861° E	190
	Shahpur	32.2878° N	72.4288° E	210

### DNA extraction

Genomic DNA was extracted from ∼20 mg at the anterior end of each adult fluke (*n* = 595) using the DNeasy Blood and Tissue kit (Qiagen), according to the manufacturer’s instructions, with a final elution volume of 100 µl. Assessment of DNA concentration and quality was carried out using a NanoDrop One Spectrophotometer (Thermo Fisher Scientific, UK).

DNA was also extracted using the same method from known samples of *F. hepatica* and *F. gigantica* to act as positive PCR controls. *Fasciola hepatica* were recovered from cattle at local abattoirs from across Europe (Ireland, Spain, the Netherlands; Walker et al, 2011, 2012) and *F. gigantica* were collected from cattle/buffalo at local abattoirs from Cameroon, India (Walker et al, 2012), Tanzania (Walker et al, 2008), Uganda and Vietnam (Supplemental file 2).

### PCR amplification of *Fasciola* spp. markers and gel electrophoresis

PCR amplification of *pepck, fabp* and RAPD from individual adult liver fluke samples was carried out in a 20 µl reaction volume containing 2X DreamTaq Green PCR master mix (Thermo Fisher Scientific), 0.5 µM of respective primers (Table 2; Integrated DNA Technologies, IDT) and 1 μl of DNA template (average ∼300 ng). The *pepck* and *fabp* PCR protocols have been developed to include a single step where all the primers are included in the same reaction (Shoriki et al, 2016; Okamoto et al, 2022), whereas separate PCR reactions are required for the species-specific RAPD PCRs (McGarry et al, 2007). Known DNA samples of *F. hepatica* and *F. gigantica* (Walker et al, 2008, 2011, 2012; Supplemental file 2), and water, were used as positive and negative samples, respectively. The cycling conditions were an initial 3 min at 95°C, followed by 30 cycles at 95°C for 30 s, annealing temperature (see Table 2) for 30 s and 72°C for 30 s, with a final extension at 72°C for 10 min. PCR products were analysed by agarose gel electrophoresis (1% gel) using SYBR® Safe DNA stain (Thermo Fisher Scientific).

**Table 2.** *Fasciola* spp. primer sequences

Primer sequence (5ʹ−3ʹ)	Ta (°C)	References
*Pepck*		
Fh-pepck-F: GATTGCACCGTTAGGTTAGC Fg-pepck-F: AAAGTTTCTATCCCGAACGAAG Fcmn-pepck-R: CGAAAATTATGGCATCAATGGG	55	Hayashi et al, 2018
*Fabp*		
FABP_ComnF: GCGGTTCTGAGTGTGTGTTT FABP-FhR: TGACGAACAGCTTACCTTCGAG FABP_FgR: CAATACTCCTCACCACCCAG	55	Okamoto et al, 2022
RAPD		
FhepF: GCGGCCAAATATGAGTCA FhepR: CTGGAGATTCCGGTTACCAA FgigF: GTTCAGGTGACAAGCCAA FgigR: ATCACACCGTGAAGCAGA	Fh: 54 Fg: 56	McGarry et al, 2007
*mt-nd1*		
FasND1_F*: ACACTCTTTCCCTACACGACGCTCTTCCGATCTGTTTAAGTTTGTGTTTTTTC FasND1_R*: GTGACTGGAGTTCAGACGTGTGCTCTTCCGATCTCACCATAACTCCCCCAAACCA	60	This study
*fabp* check		
Fas_FABP_F: CTGGTGATGTTGAGAAGG Fg_FABP_R: AATCGTTGTTTACACCCTC	50	Okamoto et al, 2022
FABP_Clo-F: CCATTGGTTTATAATAACTTCC FABP_Clo-R: ACTTCATTTTCTCCATCCTTC	50	Okamoto et al, 2022
FABP_Clo-F: CCATTGGTTTATAATAACTTCC FABP_FgR: CAATACTCCTCACCACCCAG	50	Okamoto et al, 2022
FABP_ComnF: GCGGTTCTGAGTGTGTGTTT FABP_Clo-R: ACTTCATTTTCTCCATCCTTC	50	Okamoto et al, 2022

* Primers include recognition sequences that allowed a secondary nested PCR by CGR.

### Fabp sequence analysis

Clarification of the *fabp* sequence was carried out using a nested PCR approach on the liver fluke samples from which the *fabp* sequence could not be initially amplified, using primers designed by Okamoto et al (2022). The primary PCR was carried out in a 50 µl reaction volume containing Q5 High-Fidelity 2X master mix (New England Biolabs), 0.5 µM of respective primers (Fas_FABP_F and Fg_FABP_R; Table 2; Integrated DNA Technologies, IDT) and 1 μl of DNA template. This was followed by the nested PCR similarly carried out in a 50 µl reaction volume containing Q5 High-Fidelity 2X master mix (New England Biolabs), 0.5 µM of respective primers (FABP_Clo-F and FABP_Clo-R; Table 2; Integrated DNA Technologies, IDT) and 2 μl of the primary PCR product. PCR products were analysed by agarose gel electrophoresis (1% gel) using SYBR® Safe DNA stain (Thermo Fisher Scientific). The cycling conditions were an initial 30 s at 98°C, followed by 30 cycles at 98°C for 10 s, annealing temperature (see Table 2) for 30 s and 72°C for 1 min, with a final extension at 72°C for 2 min. PCR products were analysed by agarose gel electrophoresis (1% gel) using SYBR® Safe DNA stain (Thermo Fisher Scientific). Positive PCR products were purified using the QIAquick PCR Purification Kit (Qiagen) according to the manufacturer’s instructions, using a final elution of 40 μl. DNA concentration and quality was assessed by NanoDrop One Spectrophotometer (Thermo Fisher Scientific, UK) prior to being sent for Sanger sequencing at Eurofins Genomics. Consensus sequences were compiled for the forward and reverse Sanger sequencing reads and alignments against the *F. gigantica fabp* sequence (PRJNA230515; F_gigantica-1.0_Cont4711:50422:55108:-1) carried out using Clustal Omega.

Combinations of the Clo primers and the *fabp* marker primers (FABP_ComnF and FABP_Clo-R: 580 bp; FABP_Clo-F and FABP_FgR: 218bp) were assessed followed by a nested PCR approach using FABP_ComnF and FABP_FgR primers on the PCR products. Each PCR was carried out in a 20 µl reaction volume containing 2X DreamTaq Green PCR master mix (Thermo Fisher Scientific), 0.5 µM of respective primers and 1 μl of DNA template. The cycling conditions were an initial 3 min at 95°C, followed by 30 cycles at 95°C for 30 s, annealing temperature (see Table 2) for 30 s and 72°C for 30 s, with a final extension at 72°C for 10 min. PCR products were analysed by agarose gel electrophoresis (1% gel) using SYBR® Safe DNA stain (Thermo Fisher Scientific).

Analysis of the genomic location corresponding to the primer sequences was carried out against the *F. hepatica* and *F. gigantica* genomes using the BLAST function on WormBase ParaSite (https://parasite.wormbase.org).

### Illumina sequencing of mitochondrial NADH dehydrogenase (mt-nd1) PCR products

PCR amplification of *mt-nd1* was carried out on pooled DNA samples. Equimolar concentrations of DNA extracted from the adult liver flukes (*n* = 4–7) collected from each liver were pooled together to form a single DNA sample representing a single animal. Individual DNA samples of known *F. hepatica* and *F. gigantica* samples (Supplemental file 2) were used as positive controls.

A primary PCR amplification of *mt-nd1* was carried out in a 25 µl reaction volume containing Q5 High-Fidelity 2X master mix (New England Biolabs), 0.5 µM of respective primers (Table 2; Eurofins) and 1 μl of DNA template (average ∼130 ng). The cycling conditions were an initial 30 s at 98°C, followed by 10 cycles at 98°C for 10 s, 60°C for 30 s and 72°C for 30 s, with a final extension at 72°C for 2 min. These PCR products were then used for metabarcoding and library preparation for Illumina paired-end sequencing (2x250bp; MiSeq v2) carried by the Centre of Genomic Research (CGR, Liverpool, UK).

The raw Fastq files are trimmed for the presence of Illumina adapter sequences using Cutadapt version 1.2.1 (Martin, 2011), using the -O 3 option. The reads are further trimmed using Sickle version 1.200 with a minimum window quality score of 20. Reads shorter than 15 bp. after trimming were removed. Subsequent data analysis was carried out using the Mothur v.1.48.0 software (Schloss et al, 2009) according to the pipeline used by Rehman et al. (2020, 2021). Briefly, the paired-end reads were combined into contigs and checked for ambiguous bases (make.contigs and screen.seqs commands). *Fasciola* species identification was determined by aligning the contig sequences with *Fasciola* spp. *mt-nd1* sequences compiled from NCBI (Supplemental file 1; align.seqs). Consensus sequences for each animal sample were compiled (pre.cluster, chimera.vsearch, split.groups) and classified by *Fasciola* spp. (classify.seqs). To ensure no artefacts were present, the consensus sequences were aligned using the MUSCLE alignment tool in Geneious v2024.0.7, to identify any polymorphisms that were only present once, which were removed from the subsequent analysis. The frequency of the haplotypes present was determined by dividing the number of sequence reads per haplotype by the total number of reads; the most dominant haplotypes representing > 5% frequency were used for subsequent analysis.

### Network and split tree analysis

Duplicate *mt-nd1* haplotype sequences generated in this study compared with those reported by Rehman et al. (2020, 2021) were collapsed into representative sequences using FaBox (v 1.61; Villesen, 2007). The resulting sequences were used for network analysis carried out using PopART (Population Analysis with Reticulate Trees; Leigh and Bryant, 2015) based on Median Joining Network. Split trees were created using the SplitsTree App (Huson and Bryant, 2024) using Jukes Cantor Distance within a neighbour network. Analysis of the single nucleotide polymorphisms (SNP) were performed on alignments of the haplotype sequences carried out using Clustal Omega.

## Results

### Gel-based markers

Assessment of *Fasciola* spp. was carried out based on at least two gel-based markers (*pepck, fabp* and RAPD) for all the 595 adult liver fluke samples collected across the four main provinces in Pakistan (Supplemental file 3). For each of the markers, consistent bands to that previously reported (McGarry et al, 2007; Shoriki et al, 2016; Okamoto et al, 2022) were observed. Positive bands associated with *F. gigantica* were observed for all the 595 samples using the *pepck* PCR, independent of host species or location. This observation was confirmed using the *fabp* and RAPD markers; however, inconsistent results were observed using the *fabp* PCR protocol. Specifically, *fabp* PCR products associated with *F. gigantica* could only be amplified from 321 samples (54%; Supplemental file 3). The remaining samples (274 parasites; 46%) were negative, indicating potential sequence differences in the *fabp* primer sequences. The negative PCR results were not associated with a specific host nor geographical location, with adult liver fluke samples recovered from an individual animal displaying both positive and negative *fabp* PCR amplification (Supplemental file 3). The *Fasciola* species classification of the 274 samples negative in the *fabp* PCR was further assessed using the RAPD PCR. For each sample, RAPD PCR products were amplified corresponding to *F. gigantica*, further confirming the species of the adult flukes recovered from the cattle and buffalo.

### Assessment of fabp sequence

To determine whether primer mis-priming was the cause of the negative *fabp* PCR amplification, a subset of samples representing a cross-section of locations across the 4 provinces (54; 19.7% of 274) were used for a nested PCR approach using primer sets that amplified a larger fragment of the *fabp* intron sequence (Okamoto et al, 2022). Amplification of the *fabp* sequence using the FABP_Clo-F and FABP_Clo-R primers was successful for 35 of the 54 samples. Sanger sequencing of a subset of the positive PCR products revealed that the sequences corresponding to the *fabp* primers (Fas_FABP_F and Fg_FABP_R) were identical, with the exception of one sequence that had a single-nucleotide polymorphism within the primer sequence which should not have negatively impacted the primer binding (Supplemental file 4). This result indicates that polymorphisms within the primer sequence were not the cause for the negative PCR result. BLAST analysis of the *fabp* primer sequences against the *F. gigantica* genome (PRJNA230515) indicates that there are potentially several locations within the genome with comparable sequences to our primers of interest (Supplemental file 5), which may be resulting in mis-priming and subsequent negative PCR reactions.

Combinations of the primers reported by Okamoto et al (2022), namely the Clo primers and the *fabp* marker primers, were used to assess whether one primer of the pair was influencing the potential mis-priming. Both primer combinations resulted in several bands inconsistent with the expected size, however, the nested PCR approach resulted in a strong positive band of the correct size for the *fabp* marker (Supplemental file 6). This result further supports our sequencing analysis that sequences corresponding to the *fabp* primers are present within the DNA samples, but that initial priming is inhibited in these samples.

### Sequence analysis of mt-nd1 amplicons

Amplification of *mt-nd1* was carried out on adult fluke DNA samples representing individual animals, comprised of DNA extracted from between 4 and 7 parasites. A total of 100 samples were sequenced corresponding to 18 animals from Baluchistan (buffalo *n* = 16, cattle *n* = 2), 36 animals from Punjab (buffalo *n* = 23, cattle *n* = 13), 33 animals from KPK (buffalo *n* = 15, cattle *n* = 18) and 13 animals in Gilgit Baltistan (buffalo *n* = 11, cattle *n* = 2). Based on the *Fasciola* spp. *mt-nd1* sequences compiled from NCBI, all the 100 samples sequenced were classified as *F. gigantica*, consistent with the gel-based markers. However, it should be noted that for 5 of the samples from buffalo (*n* = 4: Baluchistan–Pishin; KPK–Nowshehra and Punjab–Shahpur) and cattle (*n* = 1: Punjab–Sargodha), over 10% of the sequencing reads could only be classified to Family level (Fasciolidae). Similar inconsistencies were observed for the control *F. hepatica* and *F. gigantica* samples used for the sequence analysis. The sequencing reads from *F. gigantica* samples collected from areas in Africa where only *F. gigantica* is present (Mas-Coma et al, 2009) could not be classified to species level in 4 cases or in one sample were classified as *F. hepatica*.

**Figure 2.**
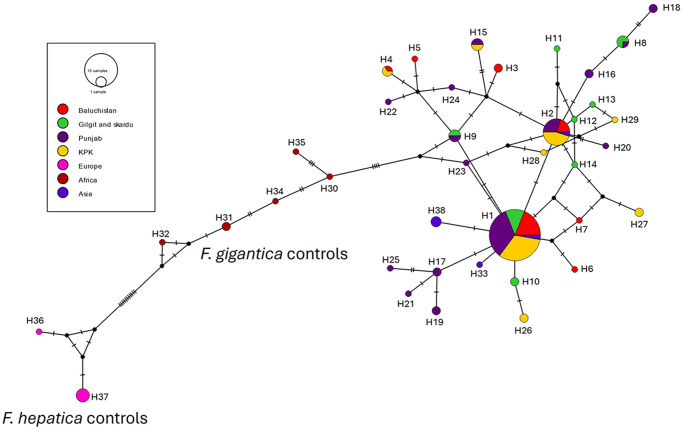
Median joining haplotype network of the *Fasciola* spp. *mt-nd1* sequences generated from the adult fluke samples from Pakistan, compared with known samples of *F. hepatica* and *F. gigantica*. The network graph was created using popart v.1.7 (http://popart.Otago.Ac.Nz). The frequency of each haplotype is represented by the size of the circles and the colour highlights from which geographical area the adult fluke sample originated. The number of mutations observed within the sequences is shown by the hatch marks, along the lines between each haplotype that is proportional to the number of base substitutions, and the unsampled/hypothetical haplotypes are shown by the black dots.

**Figure 3.**
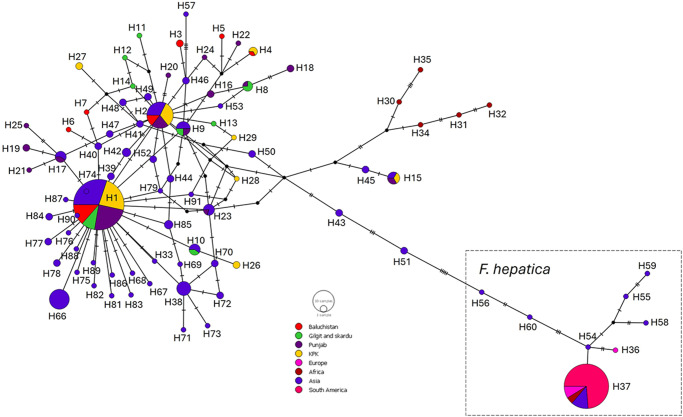
Median joining haplotype network of *Fasciola* spp. *mt-nd1* sequences. This network graph created using popart v.1.7 (http://popart.otago.ac.nz) represents all the *mt-nd1* sequences generated in this study (samples from Pakistan and positive control samples) and *Fasciola* spp. *mt-nd1* reference sequences from NCBI. The frequency of each haplotype is represented by the size of the circles and the colour highlights from which geographical area the adult fluke sample originated. The number of mutations observed within the sequences is shown by the hatch marks, along the lines between each haplotype that is proportional to the number of base substitutions, and the unsampled/hypothetical haplotypes are shown by the black dots.

Haplotype analysis identified 29 specific *mt-nd1* sequences from the 100 samples collected across the four provinces in Pakistan (Figures 2,3; Supplemental file 7). One haplotype sequence was identified in the majority of the samples (65%), with 33 samples displaying 2 haplotype sequences and 2 samples with 3 haplotype sequences. This highlights that although the DNA sample was a representative sample of a pool of between 4 and 7 parasites, the parasites recovered from a single animal all displayed similar haplotype sequences. Of these sequences, only one sequence (H1) was found in all four locations and was the predominant haplotype, present in over 69% of the samples. This sequence was also present in 2 *F. gigantica* samples from India. A further 5 haplotype sequences were present in more than one province location (H2, H4, H8, H9, H15), with haplotype H2 also present a *F. gigantica* sample from India. The remaining sequences were only present in one geographical location in a maximum of two animals.

There was considerable overlap between the sequences from the 2 Rehman et al. (2020, 2021) studies, consistent with the sample collection sites used. Five haplotype sequences (H1, H2, H15, H17, H23) are shared with the sequence data reported by Rehman et al. (2020, 2021) from adult *F. gigantica* parasites collected from across the Balochistan and Punjab provinces. This is consistent with our data that haplotype sequences H1, H2 and H15 were found across the 4 provinces sampled.

Known samples of *F. hepatica* collected from Europe and *F. gigantica* from Africa and Asia were used as positive controls. Split-tree analysis of the positive controls together with the samples from Pakistan, demonstrated clear separation of the *Fasciola* spp. sequences, with the *F. hepatica* samples represented by 2 haplotype sequences that are separated from the *F. gigantica* sequences. The *F. gigantica* from Africa form a separate group from the sequences from Pakistan and Vietnam. Based on the sequences generated as part of this study, together with the sequences from Rehman et al. (2020, 2021) and the reference *Fasciola* spp. *mt-nd1* sequences from NCBI, 64 polymorphic nucleotide positions were observed across the 309 bp sequence analysed. Nineteen polymorphic nucleotides were observed within the *F. hepatica* sequences; however, all 19 were also observed in the sequences from *F. gigantica* samples from Pakistan (Supplemental file 8) meaning it is not possible to distinguish between the 2 *Fasciola* species based on these SNPs alone. This also highlights the need for robust positive controls of known origin and species when using molecular analyses to determine *Fasciola* at the species level.

## Discussion

In this study, 4 molecular markers were evaluated to explore liver fluke species and heterogeneity in parasites collected across 4 provinces in Pakistan. While it has been previously reported that both *F. hepatica* and *F. gigantica* parasites are found in Pakistan (Afshan et al, 2014b; Rehman et al, 2020, 2021; Rizwan et al, 2022), only *F. gigantica* was observed in the samples collected for this study. This result may reflect the hosts from which the parasites were collected and the geographical location across Pakistan. In this study, liver fluke parasites were collected from buffalo and cattle, whereas the predominance of reports of *F. hepatica* has been from sheep and goats (Rehman et al, 2021). While Balochistan has been previously a source of *F. hepatica* parasites, these parasites were collected from Murgha Kibzai and Loralai regions of Balochistan, 2 areas not assessed in this study. However, our study is consistent with the dominance of *F. gigantica* across the Punjab province (Khan et al, 2009; Afshan et al, 2014b). It has also been reported that restricted by the snail intermediate host, *F. hepatica* is found in the highland regions of Pakistan compared to *F. gigantica* that is found in lowland regions (Kendall, 1954, 1970; Kendall and Parfitt, 1959; Afshan et al, 2014b). The identification of *F. hepatica* in Murgha Kibzai and Loralai, both highland areas, is consistent with these observations.

Initial studies of *Fasciola* species identification and prevalence were based on high copy number targets such as the intergenic spacer region (ITS2) and mitochondrial genes (*cox-1* and *nd-1*) (Mas-Coma et al, 2009; Hodgkinson et al, 2013; Cwiklinski et al, 2022). However, inconsistent results derived from aspermic/intermediate *Fasciola* spp. forms as a consequence of recombination and instability within the ITS region initiated the development of other markers for *Fasciola* identification (Shoriki et al, 2016). Based on single-copy nuclear genes, the *pepck* and DNA polymerase delta (*pold*) markers were observed to be more robust markers not prone to recombination events (Shoriki et al, 2016) and have since been used extensively in *Fasciola* classification studies. However, these markers are not infallible and inconsistencies/errors in the *pepck* and *pold* assays have been reported (Okamoto et al, 2022). Therefore, to add to the repertoire of *Fasciola* spp. molecular markers, Okamoto et al (2022) developed a new nuclear marker for *Fasciola* speciation based on a fatty acid binding protein (*fabp*). Utilizing comparable *Fasciola* liver fluke samples that had been used for the validation of the other markers developed by the same research group (Shoriki et al, 2016; Hayashi et al, 2018), Okamoto et al (2022) reported no discrimination errors, indicating that the *fabp* marker could be a robust marker for *Fasciola* species identification. To date, one study has been published using the *fabp* marker for species determination of samples of liver fluke in Türkiye (Celik et al, 2025).

While samples from Pakistan were included in the original validation study by Okamoto et al (2022), only 49 samples from the Punjab province were tested (Rehman et al, 2021; Okamoto et al, 2022), compared to the 595 liver fluke samples analysed in this study from across Pakistan. Despite all the liver fluke samples analysed herein (595) being classified as *F. gigantica* by other markers, the amplification of the *fabp* sequence was inconsistent in its performance. Further in-depth analysis did reveal mis-priming events could be occurring within the current multiplex primer sequences, with multiple genomic locations displaying comparable sequences, leading to the negative PCR results. A nested PCR approach did show that the *fabp* marker region of interest can be amplified, which could be used if negative results are initially generated using this marker. However, such an approach is not viable when large numbers of samples are to be tested in terms of time and cost of laboratory consumables. Therefore, it would be prudent to not solely rely on the *fabp* marker.

To delve into the *F. gigantica* population substructures within our samples from Pakistan molecular characterization of the mitochondrial NADH dehydrogenase (*mt-nd1*) was carried out that has been used in several studies (Loeurng et al, 2019; Thang et al, 2019; Rehman et al, 2020, 2021; Nazari et al, 2023). Our analysis revealed 29 *mt-nd1* haplotype sequences, of which only a small number were shared by parasites recovered from different animals from different geographical locations. In particular, the pooled liver fluke DNA samples did not display large numbers of haplotypes, indicating that the parasites were of the same/similar genetic origin. While supporting the high genetic variability observed for *Fasciola* spp. parasites in general, our data indicate that infections of individual animals are likely the result of ingestion of metacercariae from distinct populations. As farming practices across Pakistan typically consist of small holdings, this would imply that animals are being infected by liver fluke populations within their local areas, and extensive movement of livestock (facilitating parasite mixing) is not common practice.

The H1 haplotype sequence was the dominant sequence within the samples collected across Pakistan. An overlap with the haplotype sequences from Rehman et al. (2020, 2021) was observed, consistent with the comparable collection sites across the Balochistan and Punjab provinces. In comparison with the reference *F. gigantica mt-nd1* sequences from NCBI, the haplotype sequences generated in this study were most comparable to the sequences from Asia. Of the 6 haplotypes found in multiple provinces in Pakistan, 3 haplotype sequences (H1, H2 and H9) have also been reported in *F. gigantica* samples from across Asia (Bangladesh, India, Iran, Myanmar, Nepal and Thailand). In contrast, the samples from Africa displayed a different haplotype profile, with each parasite represented by a single haplotype sequence that was not observed for the liver flukes of Asian origin.

While several *F. hepatica* sequences were recovered from NCBI, collectively the sequences generated in this study together with those in NCBI, collapsed down to two haplotype sequences. These results potentially indicate lower levels of variability within the *mt-nd1* sequence within *F. hepatica* populations. However, it should also be noted that in this study *F. gigantica* was a greater focus due to the classification of the parasites all being *F. gigantica*, so the number of *F. hepatica* samples included was not as extensive.

Illumina sequencing of the PCR amplified *mt-nd1* products generates large amounts of sequence data per sample. In this study, the focus was the most dominant haplotype sequences representing > 5% frequency of the total number of sequence reads generated per sample after processing using the Mothur pipeline (68,000 average number sequence reads/sample). This approach was taken as haplotype sequences represented by a low number of sequence reads could be as a result of sequencing errors. Identification of rare haplotype sequences was also not a focus of this study. Part of the Mothur pipeline utilizes reference sequences to taxonomically classify the sequences. While all the sequences analysed, including the control reference parasites used in the study, could be classified to Family level (Fasciolidae), there were several sequences that could not be determined to species level or in one case was incorrectly classified as *F. hepatica* rather than *F. gigantica*. Our analysis also revealed that despite 64 polymorphic nucleotide positions being identified across all the sequences included in the *mt-nd1* analysis, no SNPs were specific to one *Fasciola* species. Specifically, all the SNPs identified in the *F. hepatica* sequences were also identified across the *F. gigantica* sequences. Therefore, this type of analysis requires robust reference sequences of known origin to accurately classify the haplotype sequences to species level.

While there are benefits in using this range of molecular markers for analysis of *Fasciola* spp. populations, this study highlights that inconsistencies in the resulting data can occur. Similarly, studies of *Fasciola* spp. hybrids using more than one molecular marker have shown that potential hybridization between the two *Fasciola* spp. is not uniform across the genome, with mitochondrial markers displaying different results compared to ribosomal and nuclear markers (Ichikawa-Seki et al, 2017; Nukeri et al, 2022). Therefore, it can be argued that robust analysis of *Fasciola* species identification requires several molecular markers spread throughout the genome, together with positive controls of known origin, as exemplified by the recent studies by Nazari et al (2023) and Hecker et al (2025) that also used microsatellite markers. Developments in sequencing technologies are also facilitating whole-genome sequencing approaches that could be employed on specific samples of interest where sufficient parasite material was recovered. The available *Fasciola* molecular assays should be evaluated regularly using large numbers of liver fluke parasites from throughout the world to check their reliability and consistency, in line with the conclusions by Okamoto et al (2022). Importantly, efforts should continue to develop novel markers of interest that can be used by the liver fluke research community.

Overall, the predominance of *F. gigantica* in cattle and buffalo indicates that there may be propensity for distinct host species to harbour different parasite species and several ruminant hosts should be assessed when considering epidemiological studies of *Fasciola* spp. in Pakistan. The *mt-nd1* marker was shown to be a robust marker for investigating the substructures of liver fluke populations and highlights the high levels of *F. gigantica* genetic diversity currently circulating in cattle and buffalo in Pakistan. These data further our understanding of the evolution of these parasites and their propensity to change and adapt to their surroundings.

## Supporting information

Komal et al. supplementary material Komal et al. supplementary material

## Supplementary material

The supplementary material for this article can be found at https://doi.org/10.1017/S0031182025100693.
