# Supplementary material for: Multi-marker analysis of Fasciola gigantica from cattle and buffalo across Pakistan reveals high levels of genetic diversity and novel haplotypes
Source: Parasitology. 2025 Aug 8;152(10):1047–56. doi: 10.1017/S0031182025100693 (PMC12644955; doi:10.1017/S0031182025100693)
Supplement: Komal et al. supplementary material [file S0031182025100693sup001.zip › Supplemental file 4.pdf]

Supplemental File 4

Figure S1. Clustal Omega alignment of the *fabp* sequence amplified using the FABP_Clo-F and FABP_Clo-R primers from a subset of the liver fluke samples from Pakistan compared with the *F. gigantica* fabp genome sequence (*Fg_fabp*; PRJNA230515). The location of the *fabp* marker primers (FABP_ComnF and FABP_FgR) are highlighted in yellow. Nucleotide base pair identity is shown by the asterisk. B: Baluchistan; G: Gilgit Baltistan; K: KPK; P: Punjab.

```
Fg_fabp       ATAACTTCCATTTAGTGCGGTTCTGAGTGTGTGTTTGTGTACTCAAAAACATGTGCATTG  60
B: PK-105.6   ATAACTTCCATTTAGTGCGGTTCTGAGTGTGTGTTTGTGTACTCAAAAACATGTGCATTG  60
P: PK-30.1    ATAACTTCCATTTAGTGCGGTTCTGAGTGTGTGTTTGTGTACTCAAAAACATGTGCATTG  60
K: PK-34.3    ATAACTTCCATTTAGTGCGGTTCTGAGTGTGTGTTTGTGTACTCAAAAACATGTGCATTG  60
P: PK-38.1    ATAACTTCCATTTAGTGCGGTTCTGAGTGTGTGTTTGTGTACTCAAAAACATGTGCATTG  60
K: PK-46.1    ATAACTTCCATTTAGTGCGGTTCTGAGTGTGTGTTTGTGTACTCAAAAACATGTGCATTG  60
P: PK-1.4     ATAACTTCCATTTAGTGCGGTTCTGAGTGTGTGTTTGTGTACTCAAAAACATGTGCATTG  60
P: PK-64.5    ATAACTTCCATTTAGTGCGGTTCTGAGTGTGTGTTTGTGTACTCAAAAACATGTGCATTG  60
K: PK-70.1    ATAACTTCCATTTAGTGCGGTTCTGAGTGTGTGTTTGTGTACTCAAAAACATGTGCATTG  60
K: PK-86.3    ATAACTTCCATTTAGTGCGGTTCTGAGTGTGTGTTTGTGTACTCAAAAACATGTGCATTG  60
K: PK-99.2    ATAACTTCCATTTAGTGCGGTTCTGAGTGTGTGTTTGTGTACTCAAAAACATGTGCATTG  60
P: PK-103.3   ATAACTTCCATTTAGTGCGGTTCTGAGTGTGTGTTTGTGTACTCAAAAACATGTGCATTG  60
P: PK-11.3    ATAACTTCCATTTAGTGCGGTTCTGAGTGTGTGTTTGTGTACTCAAAAACATGTGCATTG  60
G: PK-117.4   ATAACTTCCATTTAGTGCGGTTCTGAGTGTGTGTTTGTGTACTCAAAAACATGTGCATTG  60
B: PK-124.5   ATAACTTCCATTTAGTGCGGTTCTGAGTGTGTGTTTGTGTACTCAAAAACATGTGCATTG  60
G: PK-27.3    ATAACTTCCATTTAGTGCGGTTCTGAGTGTGTGTTTGTGTACTCAAAAACATGTGCATTG  60
B: PK-57.1    ATAACTTCCATTTAGTGCGGTTCTGAGTGTGTGTTTGTGTACTCAAAAACATGTGCATTG  60
P: PK-127.6   ATAACTTCCATTTAGTGCGGTTCTGAGTGTGTGTTTGTGTACTCAAAAACATGTGCATTG  60
P: PK-23.3    ATAACTTCCATTTAGTGCGGTTCTGAGTGTGTGTTTGTGTACTCAAAAACATGTGCATTG  60
              ************************************************************

Fg_fabp       ACTGCACACATTAAAACGTCGATATTGCTGAGGAGCTCTTAAACAACTAAGGTTACACAT  120
B: PK-105.6   ACTGCACACATTAAAACGTCGATATTGCTGAGGAGCTCTTAAACAACTAAGGTTACACAT  120
P: PK-30.1    ACTGCACACATTAAAACGTCGATATTGCTGAGGAGCTCTTAAACAACTAAGGTTACACAT  120
K: PK-34.3    ACTGCACACATTAAAACGTCGATATTGCTGAGGAGCTCTTAAACAACTAAGGTTACACAT  120
P: PK-38.1    ACTGCACACATTAAAACGTCGATATTGCTGAGGAGCTCTTAAACAACTAAGGTTACACAT  120
K: PK-46.1    ACTGCACACATTAAAACGTCGATATTGCTGAGGAGCTCTTAAACAACTAAGGTTACACAT  120
P: PK-1.4     ACTGCACACATTAAAACGTCGATATTGCTGAGGAGCTCTTAAACAACTAAGGTTACACAT  120
P: PK-64.5    ACTGCACACATTAAAACGTCGATATTGCTGAGGAGCTCTTAAACAACTAAGGTTACACAT  120
K: PK-70.1    ACTGCACACATTAAAACGTCGATATTGCTGAGGAGCTCTTAAACAACTAAGGTTACACAT  120
K: PK-86.3    ACTGCACACATTAAAACGTCGATATTGCTGAGGAGCTCTTAAACAACTAAGGTTACACAT  120
K: PK-99.2    ACTGCACACATTAAAACGTCGATATTGCTGAGGAGCTCTTAAACAACTAAGGTTACACAT  120
P: PK-103.3   ACTGCACACATTAAAACGTCGATATTGCTGAGGAGCTCTTAAACAACTAAGGTTACACAT  120
P: PK-11.3    ACTGCACACATTAAAACGTCGATATTGCTGAGGAGCTCTTAAACAACTAAGGTTACACAT  120
G: PK-117.4   ACTGCACACATTAAAACGTCGATATTGCTGAGGAGCTCTTAAACAACTAAGGTTACACAT  120
B: PK-124.5   ACTGCACACATTAAAACGTCGATATTGCTGAGGAGCTCTTAAACAACTAAGGTTACACAT  120
G: PK-27.3    ACTGCACACATTAAAACGTCGATATTGCTGAGGAGCTCTTAAACAACTAAGGTTACACAT  120
B: PK-57.1    ACTGCACACATTAAAACGTCGATATTGCTGAGGAGCTCTTAAACAACTAAGGTTACACAT  120
P: PK-127.6   ACTGCACACATTAAAACGTCGATATTGCTGAGGAGCTCTTAAACAACTAAGGTTACACAT  120
P: PK-23.3    ACTGCACACATTAAAACGTCGATATTGCTGAGGAGCTCTTAAACAACTAAGGTTACACAT  120
              ************************************************************

Fg_fabp       CATGGGGAAGTCAGAAATGTAGGGAAACCCACACCCAAGCTGCATTAGACCCCTTCTATG  180
B: PK-105.6   CATGGGGAAGTCAGAAATGTAGGGAAACCCACACCCAAGCTGCATTAGACCCATTCTGTG  180
P: PK-30.1    CATGGGGAAGTCAGAAATGTAGGGAAACCCACACCCAAGCTGCATTAGACCCATTCTGTG  180
K: PK-34.3    CATGGGGAAGTCAGAAATGTAGGGAAACCCACACCCAAGCTGCATTAGACCCATTCTGTG  180
P: PK-38.1    CATGGGGAAGTCAGAAATGTAGGGAAACCCACACCCAAGCTGCATTAGACCCATTCTGTG  180
K: PK-46.1    CATGGGGAAGTCAGAAATGTAGGGAAACCCACACCCAAGCTGCATTAGACCCATTCTGTG  180
P: PK-1.4     CATGGGGAAGTCAGAAATGTAGGGAAACCCACACCCAAGCTGCATTAGACCCATTCTGTG  180
P: PK-64.5    CATGGGGAAGTCAGAAATGTAGGGAAACCCACACCCAAGCTGCATTAGACCCATTCTGTG  180
K: PK-70.1    CATGGGGAAGTCAGAAATGTAGGGAAACCCACACCCAAGCTGCATTAGACCCATTCTGTG  180
K: PK-86.3    CATGGGGAAGTCAGAAATGTAGGGAAACCCACACCCAAGCTGCATTAGACCCATTCTGTG  180
K: PK-99.2    CATGGGGAAGTCAGAAATGTAGGGAAACCCACACCCAAGCTGCATTAGACCCATTCTGTG  180
P: PK-103.3   CATGGGGAAGTCAGAAATGTAGGGAAACCCACACCCAAGCTGCATTAGACCCATTCTGTG  180
P: PK-11.3    CATGGGGAAGTCAGAAATGTAGGGAAACCCACACCCAAGCTGCATTAGACCCATTCTGTG  180
G: PK-117.4   CATGGGGAAGTCAGAAATGTAGGGAAACCCACACCCAAGCTGCATTAGACCCATTCTGTG  180
B: PK-124.5   CATGGGGAAGTCAGAAATGTAGGGAAACCCACACCCAAGCTGCATTAGACCCATTCTGTG  180
G: PK-27.3    CATGGGGAAGTCAGAAATGTAGGGAAACCCACACCCAAGCTGCATTAGACCCATTCTGTG  180
B: PK-57.1    CATGGGGAAGTCAGAAATGTAGGGAAACCCACACCCAAGCTGCATTAGACCCATTCTGTG  180
P: PK-127.6   CATGGGGAAGTCAGAAATGTAGGGAAACCCACACCCAAGCTGCATTAGACCCATTCTGTG  180
```

```
P: PK-23.3    CATGGGGAAGTCAGAAATGTAGGGAAACCCACACCCAAGCTGCATTAGACCCATTCTGTG  180
              **************************************************** **** **

Fg_fabp       GACGACTGGGTGGTGAGGAGTATTGGGATTTGTAGTTAAATGAAAAACAAACATAAATCA  240
B: PK-105.6   GACGAATGGGTGGTGAGGAGTATTGGGATTTGTAGTTAAATGAAAAACAAACATAAATCA  240
P: PK-30.1    GACGACTGGGTGGTGAGGAGTATTGGGATTTGTAGTTAAATGAAAAACAAACATAAATCA  240
K: PK-34.3    GACGACTGGGTGGTGAGGAGTATTGGGATTTGTAGTTAAATGAAAAACAAACATAAATCA  240
P: PK-38.1    GACGACTGGGTGGTGAGGAGTATTGGGATTTGTAGTTAAATGAAAAACAAACATAAATCA  240
K: PK-46.1    GACGACTGGGTGGTGAGGAGTATTGGGATTTGTAGTTAAATGAAAAACAAACATAAATCA  240
P: PK-1.4     GACGACTGGGTGGTGAGGAGTATTGGGATTTGTAGTTAAATGAAAAACAAACATAAATCA  240
P: PK-64.5    GACGACTGGGTGGTGAGGAGTATTGGGATTTGTAGTTAAATGAAAAACAAACATAAATCA  240
K: PK-70.1    GACGACTGGGTGGTGAGGAGTATTGGGATTTGTAGTTAAATGAAAAACAAACATAAATCA  240
K: PK-86.3    GACGACTGGGTGGTGAGGAGTATTGGGATTTGTAGTTAAATGAAAAACAAACATAAATCA  240
K: PK-99.2    GACGACTGGGTGGTGAGGAGTATTGGGATTTGTAGTTAAATGAAAAACAAACATAAATCA  240
P: PK-103.3   GACGACTGGGTGGTGAGGAGTATTGGGATTTGTAGTTAAATGAAAAACAAACATAAATCA  240
P: PK-11.3    GACGACTGGGTGGTGAGGAGTATTGGGATTTGTAGTTAAATGAAAAACAAACATAAATCA  240
G: PK-117.4   GACGACTGGGTGGTGAGGAGTATTGGGATTTGTAGTTAAATGAAAAACAAACATAAATCA  240
B: PK-124.5   GACGACTGGGTGGTGAGGAGTATTGGGATTTGTAGTTAAATGAAAAACAAACATAAATCA  240
G: PK-27.3    GACGACTGGGTGGTGAGGAGTATTGGGATTTGTAGTTAAATGAAAAACAAACATAAATCA  240
B: PK-57.1    GACGACTGGGTGGTGAGGAGTATTGGGATTTGTAGTTAAATGAAAAACAAACATAAATCA  240
P: PK-127.6   GACGACTGGGTGGTGAGGAGTATTGGGATTTGTAGTTAAATGAAAAACAAACATAAATCA  240
P: PK-23.3    GACGACTGGGTGGTGAGGAGTATTGGGATTTGTAGTTAAATGAAAAACAAACATAAATCA  240
              ***** ******************************************************

Fg_fabp       GCAATTATAAATGAGAGCTAACTGATTTTGTACGCAAAACTCTAAAAGGTATGCTGTTCT  300
B: PK-105.6   GTAATTATAAATGAGAGCTAACTGATTTTGTACGCAAAACTCTAAAAGGTATGCTGTTCT  300
P: PK-30.1    GTAATTATAAATGAGAGCTAACTGATTTTGTACGCAAAACTCTAAAAGGTATGCTGTTCT  300
K: PK-34.3    GTAATTATAAATGAGAGCTAACTGATTTTGTACGCAAAACTCTAAAAGGTATGCTGTTCT  300
P: PK-38.1    GTAATTATAAATGAGAGCTAACTGATTTTGTACGCAAAACTCTAAAAGGTATGCTGTTCT  300
K: PK-46.1    GTAATTATAAATGAGAGCTAACTGATTTTGTACGCAAAACTCTAAAAGGTATGCTGTTCT  300
P: PK-1.4     GTAATTATAAATGAGAGCTAACTGATTTTGTACGCAAAACTCTAAAAGGTATGCTGTTCT  300
P: PK-64.5    GTAATTATAAATGAGAGCTAACTGATTTTGTACGCAAAACTCTAAAAGGTATGCTGTTCT  300
K: PK-70.1    GTAATTATAAATGAGAGCTAACTGATTTTGTACGCAAAACTCTAAAAGGTATGCTGTTCT  300
K: PK-86.3    GTAATTATAAATGAGAGCTAACTGATTTTGTACGCAAAACTCTAAAAGGTATGCTGTTCT  300
K: PK-99.2    GTAATTATAAATGAGAGCTAACTGATTTTGTACGCAAAACTCTAAAAGGTATGCTGTTCT  300
P: PK-103.3   GTAATTATAAATGAGAGCTAACTGATTTTGTACGCAAAACTCTAAAAGGTATGCTGTTCT  300
P: PK-11.3    GTAATTATAAATGAGAGCTAACTGATTTTGTACGCAAAACTCTAAAAGGTATGCTGTTCT  300
G: PK-117.4   GTAATTATAAATGAGAGCTAACTGATTTTGTACGCAAAACTCTAAAAGGTATGCTGTTCT  300
B: PK-124.5   GTAATTATAAATGAGAGCTAACTGATTTTGTACGCAAAACTCTAAAAGGTATGCTGTTCT  300
G: PK-27.3    GTAATTATAAATGAGAGCTAACTGATTTTGTACGCAAAACTCTAAAAGGTATGCTGTTCT  300
B: PK-57.1    GTAATTATAAATGAGAGCTAACTGATTTTGTACGCAAAACTCTAAAAGGTATGCTGTTCT  300
P: PK-127.6   GTAATTATAAATGAGAGCTAACTGATTTTGTACGCAAAACTCTAAAAGGTATGCTGTTCT  300
P: PK-23.3    GTAATTATAAATGAGAGCTAACTGATTTTGTACGCAAAACTCTAAAAGGTATGCTGTTCT  300
              * **********************************************************

Fg_fabp       CCATTACCTTGAGAGTGTTGTGTCTCGAATTCTGCTAAAACTCATTATTCTTCCAGGCGA  360
B: PK-105.6   CCATTACCTTTAGAGTGTTGTGTCTCGAATTCTGCTGAAACTCATTATTCTTCCAGGCGA  360
P: PK-30.1    CCATTACCTTTAGAGTGTTGTGTCTCGAATTCTGCTGAAACTCATTATTCTTCCAGGCGA  360
K: PK-34.3    CCATTACCTTTAGAGTGTTGTGTCTCGAATTCTGCTGAAACTCATTATTCTTCCAGGCGA  360
P: PK-38.1    CCATTACCTTTAGAGTGTTGTGTCTCGAATTCTGCTGAAACTCATTATTCTTCCAGGCGA  360
K: PK-46.1    CCATTACCTTTAGAGTGTTGTGTCTCGAATTCTGCTGAAACTCATTATTCTTCCAGGCGA  360
P: PK-1.4     CCATTACCTTTAGAGTGTTGTGTCTCGAATTCTGCTGAAACTCATTATTCTTCCAGGCGA  360
P: PK-64.5    CCATTACCTTTAGAGTGTTGTGTCTCGAATTCTGCTGAAACTCATTATTCTTCCAGGCGA  360
K: PK-70.1    CCATTACCTTTAGAGTGTTGTGTCTCGAATTCTGCTGAAACTCATTATTCTTCCAGGCGA  360
K: PK-86.3    CCATTACCTTTAGAGTGTTGTGTCTCGAATTCTGCTGAAACTCATTATTCTTCCAGGCGA  360
K: PK-99.2    CCATTACCTTTAGAGTGTTGTGTCTCGAATTCTGCTGAAACTCATTATTCTTCCAGGCGA  360
P: PK-103.3   CCATTACCTTTAGAGTGTTGTGTCTCGAATTCTGCTGAAACTCATTATTCTTCCAGGCGA  360
P: PK-11.3    CCATTACCTTTAGAGTGTTGTGTCTCGAATTCTGCTGAAACTCATTATTCTTCCAGGCGA  360
G: PK-117.4   CCATTACCTTTAGAGTGTTGTGTCTCGAATTCTGCTGAAACTCATTATTCTTCCAGGCGA  360
B: PK-124.5   CCATTACCTTTAGAGTGTTGTGTCTCGAATTCTGCTGAAACTCATTATTCTTCCAGGCGA  360
G: PK-27.3    CCATTACCTTTAGAGTGTTGTGTCTCGAATTCTGCTGAAACTCATTATTCTTCCAGGCGA  360
B: PK-57.1    CCATTACCTTTAGAGTGTTGTGTCTCGAATTCTGCTGAAACTCATTATTCTTCCAGGCGA  360
P: PK-127.6   CCATTACCTTTAGAGTGTTGTGTCTCGAATTCTGCTGAAACTCATTATTCTTCCAGGCGA  360
P: PK-23.3    CCATTACCTTTAGAGTGTTGTGTCTCGAATTCTGCTGAAACTCATTATTCTTCCAGGCGA  360
              ********** ************************* ************************

Fg_fabp       GATTTCCTCTATCACATTGTCGGAACTATCATACTCAACTGAAATATTTTAGATGACAAA  420
B: PK-105.6   GATTTCCTCTATCACATTGTCGGAACTATCATACTCAACTAAAATATTTTAGATGACAAA  420
P: PK-30.1    GATTTCCTCTATCACATTGTCGGAACTATCATACTCAACTAAAATATTTTAGATGACAAA  420
K: PK-34.3    GATTTCCTCTATCACATTGTCGGAACTATCATACTCAACTAAAATATTTTAGATGACAAA  420
P: PK-38.1    GATTTCCTCTATCACATTGTCGGAACTATCATACTCAACTAAAATATTTTAGATGACAAA  420
```

```
K: PK-46.1   GATTTCCTCTATCACATTGTCGGAACTATCATACTCAACTAAAATATTTTAGATGACAAA  420
P: PK-1.4    GATTTCCTCTATCACATTGTCGGAACTATCATACTCAACTAAAATATTTTAGATGACAAA  420
P: PK-64.5   GATTTCCTCTATCACATTGTCGGAACTATCATACTCAACTAAAATATTTTAGATGACAAA  420
K: PK-70.1   GATTTCCTCTATCACATTGTCGGAACTATCATACTCAACTAAAATATTTTAGATGACAAA  420
K: PK-86.3   GATTTCCTCTATCACATTGTCGGAACTATCATACTCAACTAAAATATTTTAGATGACAAA  420
K: PK-99.2   GATTTCCTCTATCACATTGTCGGAACTATCATACTCAACTAAAATATTTTAGATGACAAA  420
P: PK-103.3  GATTTCCTCTATCACATTGTCGGAACTATCATACTCAACTAAAATATTTTAGATGACAAA  420
P: PK-11.3   GATTTCCTCTATCACATTGTCGGAACTATCATACTCAACTAAAATATTTTAGATGACAAA  420
G: PK-117.4  GATTTCCTCTATCACATTGTCGGAACTATCATACTCAACTAAAATATTTTAGATGACAAA  420
B: PK-124.5  GATTTCCTCTATCACATTGTCGGAACTATCATACTCAACTAAAATATTTTAGATGACAAA  420
G: PK-27.3   GATTTCCTCTATCACATTGTCGGAACTATCATACTCAACTAAAATATTTTAGATGACAAA  420
B: PK-57.1   GATTTCCTCTATCACATTGTCGGAACTATCATACTCAACTAAAATATTTTAGATGACAAA  420
P: PK-127.6  GATTTCCTCTATCACATTGTCGGAACTATCATACTCAACTAAAATATTTTAGATGACAAA  420
P: PK-23.3   GATTTCCTCTATCACATTGTCGGAACTATCATACTCAACTAAAATATTTTAGATGACAAA  420
             **************************************** *******************

Fg_fabp      ATTTTAAACACTCGTGTTCTGAAGAGAGGTTATCTGAGCCCGACGAAACAGTATTTTTAC  480
B: PK-105.6  ATTTTAAACACTCGTGTTCTGAAGAGAGGTTATCTGAGCCCGACGAAACAGTATTTTTAC  480
P: PK-30.1   ATTTTAAACACTCGTGTTCTGAAGAGAGGTTATCTGAGCCCGACGAAACAGTATTTTTAC  480
K: PK-34.3   ATTTTAAACACTCGTGTTCTGAAGAGAGGTTATCTGAGCCCGACGAAACAGTATTTTTAC  480
P: PK-38.1   ATTTTAAACACTCGTGTTCTGAAGAGAGGTTATCTGAGCCCGACGAAACAGTATTTTTAC  480
K: PK-46.1   ATTTTAAACACTCGTGTTCTGAAGAGAGGTTATCTGAGCCCGACGAAACAGTATTTTTAC  480
P: PK-1.4    ATTTTAAACACTCGTGTTCTGAAGAGAGGTTATCTGAGCCCGACGAAACAGTATTTTTAC  480
P: PK-64.5   ATTTTAAACACTCGTGTTCTGAAGAGAGGTTATCTGAGCCCGACGAAACAGTATTTTTAC  480
K: PK-70.1   ATTTTAAACACTCGTGTTCTGAAGAGAGGTTATCTGAGCCCGACGAAACAGTATTTTTAC  480
K: PK-86.3   ATTTTAAACACTCGTGTTCTGAAGAGAGGTTATCTGAGCCCGACGAAACAGTATTTTTAC  480
K: PK-99.2   ATTTTAAACACTCGTGTTCTGAAGAGAGGTTATCTGAGCCCGACGAAACAGTATTTTTAC  480
P: PK-103.3  ATTTTAAACACTCGTGTTCTGAAGAGAGGTTATCTGAGCCCGACGAAACAGTATTTTTAC  480
P: PK-11.3   ATTTTAAACACTCGTGTTCTGAAGAGAGGTTATCTGAGCCCGACGAAACAGTATTTTTAC  480
G: PK-117.4  ATTTTAAACACTCGTGTTCTGAAGAGAGGTTATCTGAGCCCGACGAAACAGTATTTTTAC  480
B: PK-124.5  ATTTTAAACACTCGTGTTCTGAAGAGAGGTTATCTGAGCCCGACGAAACAGTATTTTTAC  480
G: PK-27.3   ATTTTAAACACTCGTGTTCTGAAGAGAGGTTATCTGAGCCCGACGAAACAGTATTTTTAC  480
B: PK-57.1   ATTTTAAACACTCGTGTTCTGAAGAGAGGTTATCTGAGCCCGACGAAACAGTATTTTTAC  480
P: PK-127.6  ATTTTAAACACTCGTGTTCTGAAGAGAGGTTATCTGAGCCCGACGAAACAGTATTTTTAC  480
P: PK-23.3   ATTTTAAACACTCGTGTTCTGAAGAGAGGTTATCTGAGCCCGACGAAACAGTATTTTTAC  480
             ************************************************************

Fg_fabp      TACGTGTGTTCACGTGCACAAATTCTTCTAAAATATTTATCGAAGAGAGATATAATGTTT  540
B: PK-105.6  TACGGGTGTTCACGTGCACAAATTCTTCTAAAATATTTATCGAAGAGAGATATAGTGTTT  540
P: PK-30.1   TACGGGTGTTCACGTGCACAAATTCTTCTAAAATATTTATCGAAGAGAGATATAGTGTTT  540
K: PK-34.3   TACGGGTGTTCACGTGCACAAATTCTTCTAAAATATTTATCGAAGAGAGATATAGTGTTT  540
P: PK-38.1   TACGGGTGTTCACGTGCACAAATTCTTCTAAAATATTTATCGAAGAGAGATATAGTGTTT  540
K: PK-46.1   TACGGGTGTTCACGTGCACAAATTCTTCTAAAATATTTATCGAAGAGAGATATAGTGTTT  540
P: PK-1.4    TACGGGTGTTCACGTGCACAAATTCTTCTAAAATATTTATCGAAGAGAGATATAGTGTTT  540
P: PK-64.5   TACGGGTGTTCACGTGCACAAATTCTTCTAAAATATTTATCGAAGAGAGATATAGTGTTT  540
K: PK-70.1   TACGGGTGTTCACGTGCACAAATTCTTCTAAAATATTTATCGAAGAGAGATATAGTGTTT  540
K: PK-86.3   TACGGGTGTTCACGTGCACAAATTCTTCTAAAATATTTATCGAAGAGAGATATAGTGTTT  540
K: PK-99.2   TACGGGTGTTCACGTGCACAAATTCTTCTAAAATATTTATCGAAGAGAGATATAGTGTTT  540
P: PK-103.3  TACGGGTGTTCACGTGCACAAATTCTTCTAAAATATTTATCGAAGAGAGATATAGTGTTT  540
P: PK-11.3   TACGGGTGTTCACGTGCACAAATTCTTCTAAAATATTTATCGAAGAGAGATATAGTGTTT  540
G: PK-117.4  TACGGGTGTTCACGTGCACAAATTCTTCTAAAATATTTATCGAAGAGAGATATAGTGTTT  540
B: PK-124.5  TACGGGTGTTCACGTGCACAAATTCTTCTAAAATATTTATCGAAGAGAGATATAGTGTTT  540
G: PK-27.3   TACGGGTGTTCACGTGCACAAATTCTTCTAAAATATTTATCGAAGAGAGATATAGTGTTT  540
B: PK-57.1   TACGTGTGTTCACGTGCACAAATTCTTCTAAAATATTTATCGAAGAGAGATATAGTGTTT  540
P: PK-127.6  TACGTGTGTTCACGTGCACAAATTCTTCTAAAATATTTATCGAAGAGAGATATAGTGTTT  540
P: PK-23.3   TACGTGTGTTCACGTGCACAAATTCTTCTAAAATATTTATCGAAGAGAGATATAGTGTTT  540
             **** ************************************************* *****

Fg_fabp      AACCGAAAACTCACTGCTGATGTTGAGAGTCGAACGAAGGAT     582
B: PK-105.6  AACCGAAAACTCACTGCTGATGTTGAGAGTCGAACGAAGGAT     582
P: PK-30.1   AACCGAAAACTCACTGCTGATGTTGAGAGTCGAACGAAGGAT     582
K: PK-34.3   AACCGAAAACTCACTGCTGATGTTGAGAGTCGAACGAAGGAT     582
P: PK-38.1   AACCGAAAACTCACTGCTGATGTTGAGAGTCGAACGAAGGAT     582
K: PK-46.1   AACCGAAAACTCACTGCTGATGTTGAGAGTCGAACGAAGGAT     582
P: PK-1.4    AACCGAAAACTCACTGCTGATGTTGAGAGTCGAACGAAGGAT     582
P: PK-64.5   AACCGAAAACTCACTGCTGATGTTGAGAGTCGAACGAAGGAT     582
K: PK-70.1   AACCGAAAACTCACTGCTGATGTTGAGAGTCGAACGAAGGAT     582
K: PK-86.3   AACCGAAAACTCACTGCTGATGTTGAGAGTCGAACGAAGGAT     582
K: PK-99.2   AACCGAAAACTCACTGCTGATGTTGAGAGTCGAACGAAGGAT     582
P: PK-103.3  AACCGAAAACTCACTGCTGATGTTGAGAGTCGAACGAAGGAT     582
P: PK-11.3   AACCGAAAACTCACTGCTGATGTTGAGAGTCGAACGAAGGAT     582
```

```
G: PK-117.4  AACCGAAAACTCACTGCTGATGTTGAGAGTCGAACGAAGGAT     582
B: PK-124.5  AACCGAAAACTCACTGCTGATGTTGAGAGTCGAACGAAGGAT     582
G: PK-27.3   AACCGAAAACTCACTGCTGATGTTGAGAGTCGAACGAAGGAT     582
B: PK-57.1   AACCGAAAACTCACTGCTGATGTTGAGAGTCGAACGAAGGAT     582
P: PK-127.6  AACCGAAAACTCACTGCTGATGTTGAGAGTCGAACGAAGGAT     582
P: PK-23.3   AACCGAAAACTCACTGCTGATGTTGAGAGTCGAACGAAGGAT     582
             ******************************************
```
