# Supplementary material for: Multi-marker analysis of Fasciola gigantica from cattle and buffalo across Pakistan reveals high levels of genetic diversity and novel haplotypes
Source: Parasitology. 2025 Aug 8;152(10):1047–56. doi: 10.1017/S0031182025100693 (PMC12644955; doi:10.1017/S0031182025100693)

Supplemental File 6

Figure S2. *fabp* sequence analysis in samples originally negative for the *fabp* marker. Amplification of *fabp* sequence using (A) FABP_Clo-F and FABP_Clo-R primers and (B) Nested approach using Fas_FABP_F and Fg_FABP_R primers on the PCR reactions used in panel A.

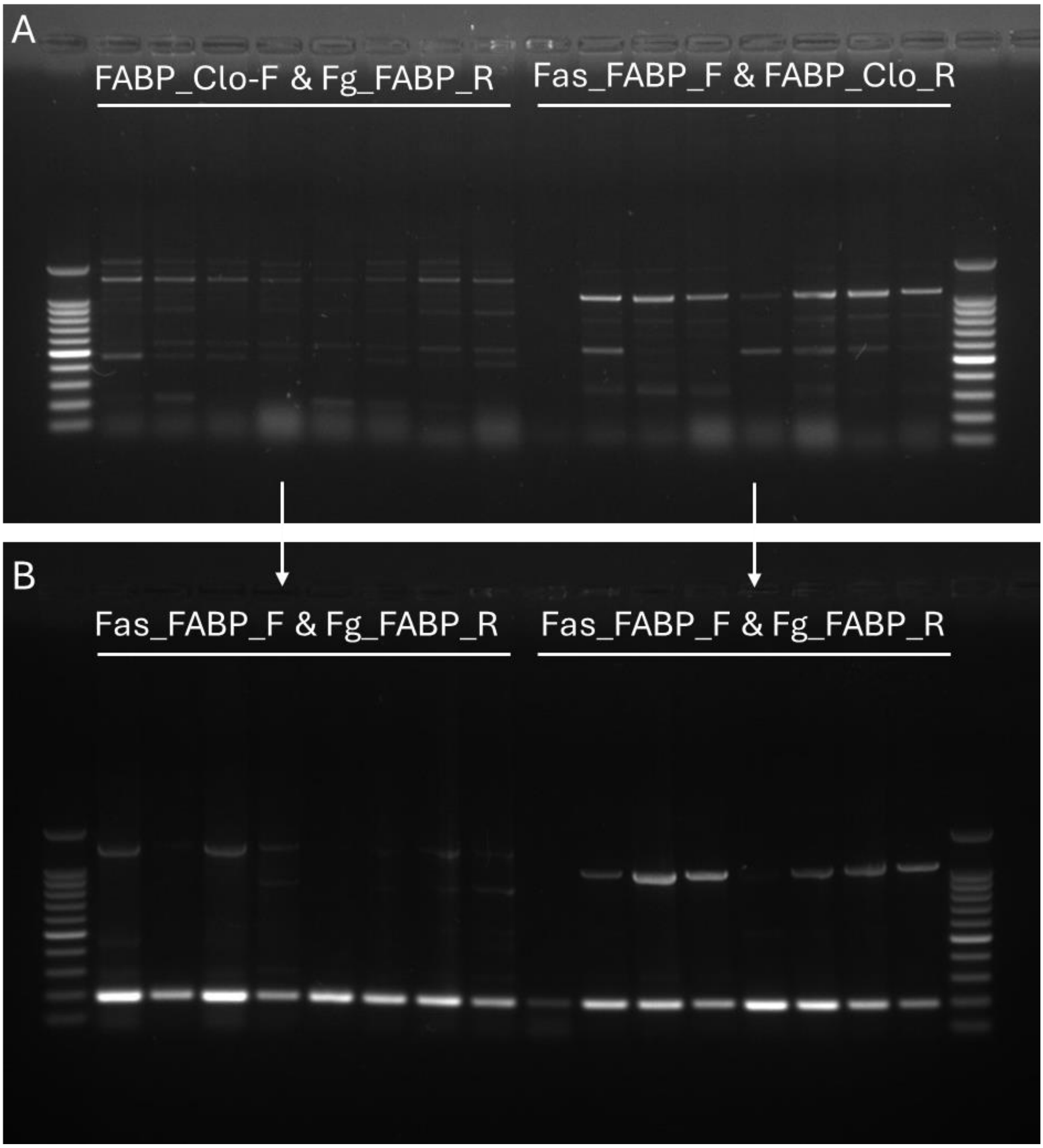

Supplement: Komal et al. supplementary material [file S0031182025100693sup001.zip › Supplemental File 6.pdf]
