# Supplementary material for: Multi-marker analysis of Fasciola gigantica from cattle and buffalo across Pakistan reveals high levels of genetic diversity and novel haplotypes
Source: Parasitology. 2025 Aug 8;152(10):1047–56. doi: 10.1017/S0031182025100693 (PMC12644955; doi:10.1017/S0031182025100693)

Supplemental File 7

Figure S3. Split tree of the *Fasciola* spp. *mt-nd1* sequences generated from the adult fluke samples from Pakistan, constructed using the SplitsTree App using Jukes Cantor Distance within a neighbour network. The tree is comparable to the median joining haplotype network displayed in Fig 2.

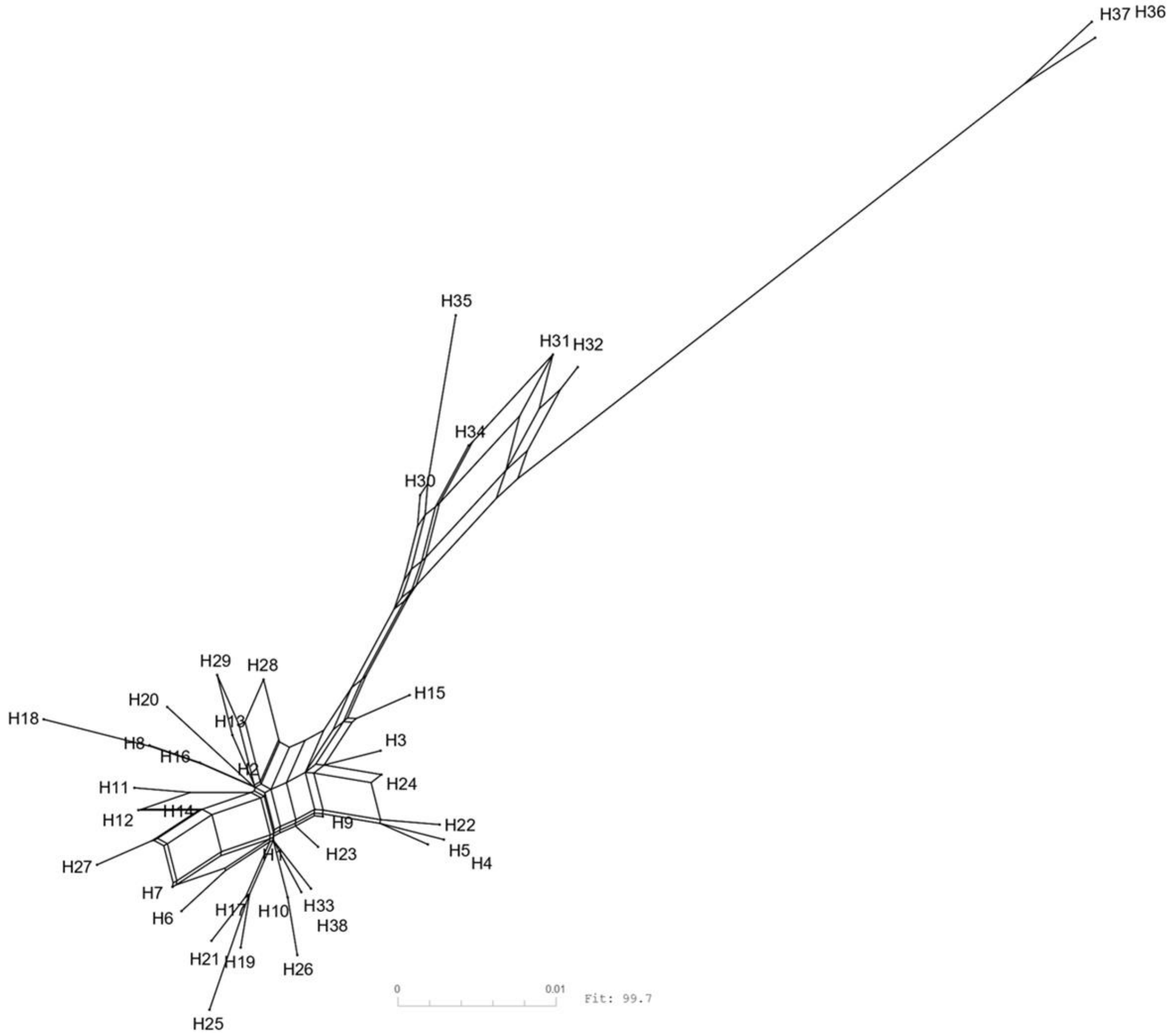

Figure S4. Split tree of *Fasciola* spp. *mt-nd1* sequences, constructed using the SplitsTree App using Jukes Cantor Distance within a neighbour network. The tree is comparable to the median joining haplotype network displayed in Fig 3.

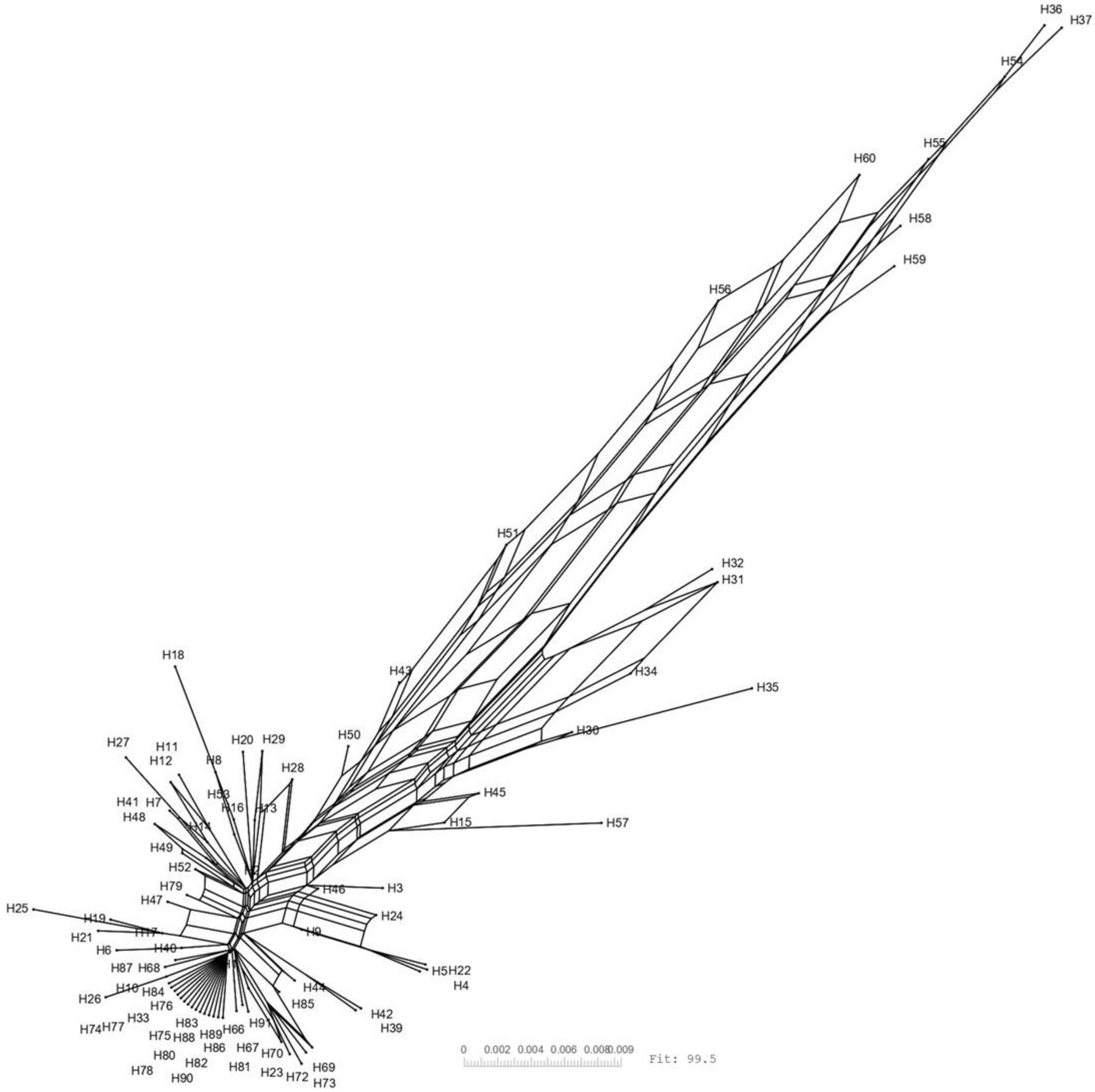

Supplement: Komal et al. supplementary material [file S0031182025100693sup001.zip › Supplemental File 7.pdf]
