# Supplementary material for: Multi-marker analysis of Fasciola gigantica from cattle and buffalo across Pakistan reveals high levels of genetic diversity and novel haplotypes
Source: Parasitology. 2025 Aug 8;152(10):1047–56. doi: 10.1017/S0031182025100693 (PMC12644955; doi:10.1017/S0031182025100693)
Supplement: Komal et al. supplementary material [file S0031182025100693sup001.zip › Supplemental File 8.pdf]

Supplemental File 8

Figure S5. Clustal Omega alignment of the mt-nd1 haplotype sequences, corresponding to Supplemental File 2. The variable sites are highlighted in grey. The *F. hepatica* sequences (H36 and H37) are highlighted in yellow and italics.

```
H54         GTTTAAGTTTGTGTTTTTTCAGAATCGTAGTTGGTTGTCTTGGTGGGGTGTTTATTTGTT    60
H60         GTTTAAGTTTGTGTTTTTTCAGAATCGTAGTTGGTTGTCTTGGTGGGGTGTTTATTTGTT    60
H58         GTTTAAGTTTGTGTTTTTTCAGAATCGTAGTTGGTTGTCTTGGTGGGGTGTTTATTTGTT    60
H37         GTTTAAGTTTGTGTTTTTTCAGAATCGTAGTTGGTTGTCTTGGTGGGGTGTTTATTTGTT    60
H56         GTTTAAGTTTGTGTTTTTTCAGAATCGTAGTTGGTTGTCTTGGTGGGGTGTTTATTTGTT    60
H59         GTTTAAGTTTGTGTTTTTTCAGAATCGTAGTTGGTTGTCTTGGTGAGGGGTTTATTTGTT    60
H32         GTTTAAGTTTGTGTTTTTTCAGAATCGTAGTTGGTTGTCTTGGTGGGGTGTTTATTTGTT    60
H31         GTTTAAGTTTGTGTTTTTTCAGAATCGTAGTTGGTTGTCTTGGTGGGGTGTTTATTTGTT    60
H55         GTTTAAGTTTGTGTTTTTTCAGAATCGTAGTTGGTTGTCTTGGTGGGGTGTTTATTTGTT    60
H51         GTTTAAGTTTGTGTTTTTTCAGAATCGTAGTTGGTTGTCTTGGTGGGGTGTTTATTTGTT    60
H35         GTTTAAGTTTGTGTTTTTTCAGAATCGTAGTTGGTTGTCTTGGTGGGGTGTTTATTTGTT    60
H36         GTTTAAGTTTGTGTTTTTTCAGAATCGTAGTTGGTTGTCTTGGTGGGGTGTTTATCTGTT    60
H34         GTTTAAGTTTGTGTTTTTTCAGAATCGTAGTTGGTTGTCTTGGTGGGGTGTTTATTTGTT    60
H57         GTTTAAGTTTGTGTTTTTTCAGAATCGTAGTTGGTTGTCTTGGTGAGGGGTTTATTTGTT    60
H45         GTTTAAGTTTGTGTTTTTTCAAAATCGTAGTTGGTTGTCTTGGTGGGGTGTTTATTTGTT    60
H43         GTTTAAGTTTGTGTTTTTTCAGAATCGTAGTTGGTTGTCTTGGTGGGGTGTTTATTTGTT    60
H30         GTTTAAGTTTGTGTTTTTTCAGAATCGTAGTTGGTTGTCTTGGTGGGGTGTTTATTTGTT    60
H18         GTTTAAGTTTGTGTTTTTTCAGAATCGTAGTTGGTTGTCTTGGTGAGGGGTTTATTTGTT    60
H50         GTTTAAGTTTGTGTTTTTTCAGAATCGTAGTTGGTTGTCTTGGTGGGGTGTTTATTTGTT    60
H27         GTTTAAGTTTGTGTTTTTTCAGAATCGTAGTTGGTTGTCTTGGTGAGGGGTTTATTTGTT    60
H24         GTTTAAGTTTGTGTTTTTTCAGAATCGTAGTTGGTTGTCTTGGTGAGGGGTTTATTTGTT    60
H22         GTTTAAGTTTGTGTTTTTTCAGAATCGTAGTTGGTTGTCTTGGTGAGGGGTTTATTTGTT    60
H15         GTTTAAGTTTGTGTTTTTTCAAAATCGTAGTTGGTTGTCTTGGTGAGGTGTTTATTTGTT    60
H5          GTTTAAGTTTGTGTTTTTTCAGAATCGTAGTTGGTTGTCTTGGTGAGGGGTTTATTTGTT    60
H4          GTTTAAGTTTGTGTTTTTTCAGAATCGTAGTTGGTTGTCTTGGTGAGGGGTTTATTTGTT    60
H61         -GTTTAGTTTGTGTTTTTTCAGAATCGTAGTTGGTTGTCTTGGTGAGGGGTTTATTTGTT    59
H48         GTTTAAGTTTGTGTTTTTTCAGAATCGTAGTTGGTTGTCTTGGTGAGGGGTTTATTTGTT    60
H42         GTTTAAGTTTGTGTTTTTTCAGAATCGTAGTTGGTTGTCTTGGTGAGGGGTTTATTTGTT    60
H29         GTTTAAGTTTGTGTTTTTTCAGAATCGTAGTTGGTTGTCTTGGTGAGGGGTTTATTTGTT    60
H28         GTTTAAGTTTGTGTTTTTTCAGAATCGTAGTTGGTTGTCTTGGTGGGGGGTTTATTTGTT    60
H25         GTTTAAGTTTGTGTTTTTTCAGAATCGTAGTTGGTTGTCTTGGTGAGGGGTTTATTTGTT    60
H20         GTTTAAGTTTGTGTTTTTTCAGAATCGTAGTTGGTTGTCTTGGTGAGGGGTTTATTTGTT    60
H12         GTTTAAGTTTGTGTTTTTTCAGAATCGTAGTTGGTTGTCTTGGTGAGGGGTTTATTTGTT    60
H11         GTTTAAGTTTGTGTTTTTTCAGAATCGTAGTTGGTTGTCTTGGTGAGGGGTTTATTTGTT    60
H8          GTTTAAGTTTGTGTTTTTTCAGAATCGTAGTTGGTTGTCTTGGTGAGGGGTTTATTTGTT    60
H3          GTTTAAGTTTGTGTTTTTTCAGAATCGTAGTTGGTTGTCTTGGTGAGGGGTTTATTTGTT    60
H73         GTTTAAGTTTGTGTTTTTTCAGAATCGTAGTTGGTTGTCTTGGTGAGGGGTTTATTTGTT    60
H72         GTTTAAGTTTGTGTTTTTTCAGAATCGTAGTTGGTTGTCTTGGTGCGGGGTTTATTTGTT    60
H71         GTTTAAGTTTGTGTTTTTTCAGAATCGTAGTTGGTTGTCTTGGTCAGGGGTTTATTTGTT    60
H70         GTTTAAGTTTGTGTTTTTTCAGAATCGTAGTTGGTTGTCTTGGTGGGGGGTTTATTTGTT    60
H69         GTTTAAGTTTGTGTTTTTTCAGAATCGTAGTTGGTTGTCTTGGTGAGGGGTTTATTTGTT    60
H53         GTTTAAGTTTGTGTTTTTTCAGAATCGTAGTTGGTTGTCTTGGTGAGGGGTTTATTTGTT    60
H52         GTTTAAGTTTGTGTTTTTTCAGAATCGTAGTTGGTTGTCTTGGTGAGGGGTTTATTTGTT    60
H49         GTTTAAGTTTGTGTTTTTTCAGAATCGTAGTTGGTTGTCTTGGTGAGGGGTTTATTTGTT    60
H47         GTTTAAGTTTGTGTTTTTTCAGAATCGTAGTTGGTTGTCTTGGTGAGGGGTTTATTTGTT    60
H46         GTTTAAGTTTGTGTTTTTTCAGAATCGTAGTTGGTTGTCTTGGTGAGGGGTTTATTTGTT    60
H44         GTTTAAGTTTGTGTTTTTTCAGAATCGTAGTTGGTTGTCTTGGTGAGGGGTTTATTTGTT    60
H41         GTTTAAGTTTGTGTTTTTTCAGAATCGTAGTTGGTTGTCTTGGTGAGGGGTTTATTTGTT    60
H39         GTTTAAGTTTGTGTTTTTTCAGAATCGTAGTTGGTTGTCTTGGTGAGGGGTTTATTTGTT    60
H26         GTTTAAGTTTGTGTTTTTTCAGAATCGTAGTTGGTTGTCTTGGTGAGGGGTTTATCTGTT    60
H21         GTTTAAGTTTGTGTTTTTTCAGAATCGTAGTTGGTTGTCTTGGTGAGGGGTTTATTTGTT    60
```

```
H19      GTTTAAGTTTGTGTTTTTTCAGAATCGTAGTTGGTTGTCTTGGTGAGGGGTTTATTTGTT   60
H16      GTTTAAGTTTGTGTTTTTTCAGAATCGTAGTTGGTTGTCTTGGTGAGGGGTTTATTTGTT   60
H14      GTTTAAGTTTGTGTTTTTTCAGAATCGTAGTTGGTTGTCTTGGTGAGGGGTTTATTTGTT   60
H13      GTTTAAGTTTGTGTTTTTTCAGAATCGTAGTTGGTTGTCTTGGTGAGGGGTTTATTTGTT   60
H7       GTTTAAGTTTGTGTTTTTTCAGAATCGTAGTTGGTTGTCTTGGTGAGGGGTTTATTTGTT   60
H6       GTTTAAGTTTGTGTTTTTTCAGAATCGTAGTTGGTTGTCTTGGTGAGGGGTTTATTTGTT   60
H91      GTTTAAGTTTGTGTTTTTTCAGAATCGTAGTTGGTTGTCTTGGTGAGGGGTTTATTTGTT   60
H90      GTTTAAGTTTGTGTTTTTTCAGAATCGTAGTTGGTTGTCTTGGTGAGGGGTTTATTTGTT   60
H89      GTTTAAGTTTGTGTTTTTTCAGAATCGTAGTTGGTTGTCTTGGTGAGGGGTTTATTTGTT   60
H88      GTTTAAGTTTGTGTTTTTTCAGAATCGTAGTTGGTTGTCTTGGTGAGGGGTTTATTTGTT   60
H87      GTTTAAGTTTGTGTTTTTTCAGAATCGTAGTTGGTTGTCTTGGTGAGGAGTTTATTTGTT   60
H86      GTTTAAGTTTGTGTTTTTTCAGAATCGTAGTTGGTTGTCTTGGTGAGGGGTTTATTTGTT   60
H85      GTTTAAGTTTGTGTTTTTTCAGAATCGTAGTTGGTTGTCTTGGTGAGGGGTTTATTTGTT   60
H84      GTTTAAGTTTGTGTTTTTTCAGAATCGTAGTTGGTTGTCTTGGTGAGGGGTTTATTTGTT   60
H83      GTTTAAGTTTGTGTTTTTTCAGAATCGTAGTTGGTTGTCTTGGTGAGGGGTTTATTTGTT   60
H82      GTTTAAGTTTGTGTTTTTCCAGAATCGTAGTTGGTTGTCTTGGTGAGGGGTTTATTTGTT   60
H81      GTTTAAGTTTGTGTTTTTTCAGAATCGTAGTTGGTTGTCTTGGTGAGGGGTTTATTTGTT   60
H79      GTTTAAGTTTGTGTTTTTTCAGAATCGTAGTTGGTTGTCTTGGTGAGGGGTTTATTTGTT   60
H78      GTTTAAGTTTGTGTTTTTTCAGAATCGTAGTTGGTTGTCTTGGTGAGGGGTTTATTTGTT   60
H77      GTTTAAGTTTGTGTTTTTTCAGAATCGTAGTTGGTTGTCTTGGTGAGGGGTTTATTTGTT   60
H76      GTTTAAGTTTGTGTTTTTTCAGAATCGTAGTTGGTTGTCTTGGTGAGGGGTTTATTTGTT   60
H75      GTTTAAGTTTGTGTTTTTTCAGAATCGTAGTTGGTTGTCTTGGTGAGGGGTTTATTTGTT   60
H74      GTTTAAGTTTGTGTTTTTTCAGAATCGTAGTTGGTTGTCTTGGTGAGGGGTTTATTTGTT   60
H68      GTTTAAGTTTGTGTTTTTTCAGAATCGTAGTTGGTTGTCTTGGTGAGGGGTTTATTTGTT   60
H67      GTTTAAGTTTGTGTTTTTTCAGAATCGTAGTTGGTTGTCTTGGTGAGGGGTTTATTTGTT   60
H66      GTTTAAGTTTGTGTTTTTTCAGAATCGTAGTTGGTTGTCTTGGTGAGGGATTTATTTGTT   60
H40      GTTTAAGTTTGTGTTTTTTCAGAATCGTAGTTGGTTGTCTTGGTGAGGGGTTTATTTGTT   60
H38      GTTTAAGTTTGTGTTTTTTCAGAATCGTAGTTGGTTGTCTTGGTGAGGGGTTTATTTGTT   60
H33      GTTTAAGTTTGTGTTTTTTCAGAATCGTAGTTGGTTGTCTTGGTGAGGGGTTTATTTGTT   60
H23      GTTTAAGTTTGTGTTTTTTCAGAATCGTAGTTGGTTGTCTTGGTGGGGGGTTTATTTGTT   60
H17      GTTTAAGTTTGTGTTTTTTCAGAATCGTAGTTGGTTGTCTTGGTGAGGGGTTTATTTGTT   60
H10      GTTTAAGTTTGTGTTTTTTCAGAATCGTAGTTGGTTGTCTTGGTGAGGGGTTTATTTGTT   60
H9       GTTTAAGTTTGTGTTTTTTCAGAATCGTAGTTGGTTGTCTTGGTGAGGGGTTTATTTGTT   60
H2       GTTTAAGTTTGTGTTTTTTCAGAATCGTAGTTGGTTGTCTTGGTGAGGGGTTTATTTGTT   60
H63      GTTTAAGTTTGTGTTTTTTCAGAATCGTAGTTGGTTGTCTTGGTGAGGGGTTTATTTGTT   60
H80      GTTTAAGTTTGTGTTTTTTCAGAATCGTAGTTGGTTGTCTTGGTGAGGGGTTTATTTGTT   60
H65      GTTTAAGTTTGTGTTTTTTCAGAATCGTAGTTGGTTGTCTTGGTGAGGGGTTTATTTGTT   60
H64      GTTTAAGTTTGTGTTTTTTCAGAATCGTAGTTGGTTGTCTTGGTGAGGGGTTTATTTGTT   60
H1       GTTTAAGTTTGTGTTTTTTCAGAATCGTAGTTGGTTGTCTTGGTGAGGGGTTTATTTGTT   60
H62      GTTTAAGTTTGTGTTTTTTCAGAATCGTAGTTGGTTGTCTTGGTGAGGGGTTTATTTGTT   60
           ** ************* ** *********************   **  ***** ****

H54      GGTTTTGTTGGCTTGTGGCTATTGTGTGTTGTTTTTTTTTAGGTTTGGTGGTGTTAGTAG   120
H60      GGTTTTGTTGGCTTGTGGCTATTGTGTGTTGTTTTTTTTTAGGTTTGGTGGTGTTAGTAG   120
H58      GGTTTTGTTGGCTTGTGGCTATTGTGTGTTGTTTTTTTTTAGGTTTGGTGGTGTTAGTAG   120
H37      GGTTTTGTTGGCTTGTGGATATTGTGTGTTGTTTTTTTTTAGGTTCGGTGGTGTTAGTAG   120
H56      GGTTTTGTTGGCTTGTGGCTATTGTGTGTTGTTTTTTTTTAGGTTTGGTGGTGTTAGTAG   120
H59      GGTTTTGTTGGCTTGTGGCTATTGTGTGTTGTTTTTTTTTAGGTTTGGTGGTGTTAGTAG   120
H32      GGTTTTGTTGGCTTGTGGTTATTGTTTGTTGTTTTTTCTTAGGTTTGGGGGTGTTAGTGG   120
H31      GGTTTTGTTGGCTTGTGGTTATTGTTTGTTGTTTTTTCTTAGGTTTGGAGGTGTTAGTGG   120
H55      GGTTTTGTTGGCTTGTGGCTATTGTGTGTTGTTTTTTTTTAGGTTTGGTGGTGTTAGTAG   120
H51      GGTTTTGTTGGCTTGTGGCTATTGTGTGTTGTTTTTTTTTAGGTTTGGGGGTGTTAGTAG   120
H35      GGTTTTATTGGCTTGTGGTTATTGTTTGTTGTTTTTTCTTAGGTTTGGAGGTGTTAGTGG   120
H36      GGTTTTGTTGGCTTGTGGCTATTGTGTGTTGTTTTTTTTTAGGTTTGGTGGTGTTAGTAG   120
H34      GGTTTTGTTGGCTTGTGGTTATTGTTTGTTGTTTTTTCTTAGGTTTGGAGGTGTTAGTGG   120
H57      GGTTTTGTTGGCTTGTGGTTATTGTTTGTTGTTTTTTCTTAGGTTTGGGGGTGTTAGTAG   120
H45      GGTTTTGTTGGCTTGTGGTTATTGTTTGTTGTTTTTTCTTAGGTTTGGGGGTGTTAGTAG   120
```

H43	GGTTTTGTTGGCTTGTGGTTATTGTTTGTTGTTTTTTCTTAGGTTTGGGGGTGTTAGTAG	120
H30	GGTTTTGTTGGCTTGTGGTTATTGTTTGTTGTTTTTTCTTAGGTTTGGAGGTGTTAGTGG	120
H18	GGTTTTGTTGGCTTGTGGTTATTGTTTGTTGTTTTTTTTTAGGTTTGGTGGTGTTAGTAA	120
H50	GGTTTTGTTGGCTTGTGGCTATTGTTTGTTGTTTTTTCTTAGGTTTGGGGGTGTTAGTAG	120
H27	GGTTTTGTTGGCTTGTGGCTATTGTTTGTTGTTTTTTCTTAGGTTTGGGGGTGTTAGTAG	120
H24	GGTTTTGTTGGCTTGTGGTTATTGTTTGTTGTTTTTTCTTAGGTTTGGGGGTGTTAGTAG	120
H22	GGTTTTGTTGGCTTGTGGTTATTGTTTGTTGTTTTTTCTTAGGTTTGGGGGTGTTAGTAG	120
H15	GGTTTTGTTGGCTTGTGGTTATTGTTTGTTGTTTTTTCTTAGGTTTGGGGGTGTTAGTAG	120
H5	GGTTTTGTTGGATTGTGGTTATTGTTTGTTGTTTTTTCTTAGGTTTGGGGGTGTTAGTAG	120
H4	GGTTTTGTTGGCTTGTGGTTATTGTTTGTTGTTTTTTCTTAGGTTTGGGGGTGTTAGTAG	120
H61	GGTTTTGTTGGCTTGTGGTTATTGTTTGTTGTTTTTTCTTAGGTTTGGGGGTGTTAGTAG	119
H48	GGTTTTGTTGGCTTGTGGCTATTGTTTGTTGTTTTTTCTTAGGTTTGGGGGTGTTAGTAG	120
H42	GGTTTTGTTGGCTTGTGGTTATTGTTTGTTGTTTTTTCTTAGGTTTGGGGGTGTTAGTAG	120
H29	GGTTTTGTTGGCTTGTGGTTATTGTTTGTTGTTTTTTCTTAGGTTTGGGGGGGTTAGTAG	120
H28	GGTTTTGTTGGCTTGTGGTTATTGTTTGTTGTTTTTTCTTAGGTTTGGGGGGGTTAGTAG	120
H25	GGTTTTGTTGGCTTGTGGTTATTGTTTGTTGTTTTTTCTTAGGTTTGTGGGTGTTAGTAG	120
H20	GGTTTTGTTGGCTTGTGGTTATTGTTTGTTGTTTTTTCTTAGGTTGGGGGGTGTTAGTAG	120
H12	GGTTTTGTTGGCTTGTGGTTATTGTTTGTTGTTTTTTCTTAGGTTTGGGGGTGTTAGTAG	120
H11	GGTTTTGTTGGCTTGTGGTTATTGTTTGTTGTTTTTTCTTAGGTTTGGGGGTGTTAGTAG	120
H8	GGTTTTGTTGGCTTGTGGTTATTGTTTGTTGTTTTTTTTTAGGTTTGGGGGTGTTAGTAA	120
H3	GGTTTTGTTGGCTTGTGGTTATTGTTTGTTGTTTTTTCTTAGGTTTGGGGGTGTTAGTAG	120
H73	GGTTTTGTTGGCTTGTGGTTATTGTTTGTTGTTTTTTCTTAGGTTTGGGGGTGTTAGTGG	120
H72	GGTTTTGTTGGCTTGTGGTTATTGTTTGTTGTTTTTTCTTAGGTTTGGGGGTGTTAGTAG	120
H71	GGTTTTGTTGGCTTGTGGTTATTGTTTGTTGTTTTTTCTTAGGTTTGGGGGTGTTAGTAG	120
H70	GGTTTTGTTGGCTTGTGGTTATTGTTTGTTGTTTTTTCTTAGGTTTGGGGGTGTTAGTAG	120
H69	GGTTTTGTTGGCTTGTGGTTATTGTTTGTTGTTTTTTCTTAGGTTTGGGGGTGTTAGTAG	120
H53	GGTTTTGTTGGCTTGTGGTTATTGTTTGTTGTTTTTTTTTAGGTTTGGGGGTGTTAGTAG	120
H52	GGTTTTGTTGGCTTGTGGTTATTGTTTGTTGTTTTTTCTTAGGTTTGGAGGTGTTAGTAG	120
H49	GGTTTTGTTGGCTTGTGGTTATTGTTTGTTGTTTTTTCTTAGGTTTGGGGGTGTTAGTAG	120
H47	GGTTTTGTTGGCTTGTGGTTATTGTTTGTTGTTTTTTCTTAGGTTTGGGGGTGTTAGTAG	120
H46	GGTTTTGTTGGCTTGTGGTTATTGTTTGTTGTTTTTTCTTAGGTTTGGGGGTGTTAGTAG	120
H44	GGTTTTGTTGGCTTGTGGTTATTGTTTGTTGTTTTTTCTTAGGTTTGGGGGTGTTAGTAG	120
H41	GGTTTTGTTGGCTTGTGGCTATTGTTTGTTGTTTTTTCTTAGGTTTGGGGGTGTTAGTAG	120
H39	GGTTTTGTTGGCTTGTGGTTATTGTTTGTTGTTTTTTCTTAGGTTTGGGGGTGTTAGTAG	120
H26	GGTTTTGTTGGCTTGTGGTTATTGTTTGTTGTTTTTTCTTAGGTTTGGGGGTGTTAGTAG	120
H21	GGTTTTGTTGGCTTGTGGTTATTGTTTGTTGTTTTTTCTTAGGTTTGGGGGTGTTAGTAG	120
H19	GGTTTTGTTGGCTTGTGGTTATTGTTTGTTGTTTTTTCTTAGGTTTGGGGGTGTTAGTAG	120
H16	GGTTTTGTTGGCTTGTGGTTATTGTTTGTTGTTTTTTCTTAGGTTTGGGGGTGTTAGTAA	120
H14	GGTTTTGTTGGCTTGTGGTTATTGTTTGTTGTTTTTTCTTAGGTTTGGGGGTGTTAGTAG	120
H13	GGTTTTGTTGGCTTGTGGTTATTGTTTGTTGTTTTTTCTTAGGTTTGGGGGTGTTAGTAG	120
H7	GGTTTTGTTGGCTTGTGGCTATTGTTTGTTGTTTTTTCTTAGGTTTGGGGGTGTTAGTAG	120
H6	GGTTTTGTTGGCTTGTGGCTATTGTTTGTTGTTTTTTCTTAGGTTTGGGGGTGTTAGTAG	120
H91	GGTTTTGTTGGCTTGTGGTTATTGTTTGTTGTTTTTTCTTAGGTTTGGGGGGGTTAGTAG	120
H90	GGTTTTGTTGGCTTGTGGTTATTGTTTGTTGTTTTTTCTTAGGTTTGGGGGTGTTAATAG	120
H89	GGTTTTGTTGGCTTGTGGTTATTGTTTGTTGTTTTTTCTTAGGTTTGGGGGTGTTAGTAG	120
H88	GGTTTTGTTGGCTTGTGGTTATTGTTTGTTGTTTTTTCTTAGGTTTGGGGGTGTTAGTAG	120
H87	GGTTTTGTTGGCTTGTGGTTATTGTTTGTTGTTTTTTCTTAGGTTTGGGGGTGTTAGTAG	120
H86	GGTTTTGTTGGCTTGTGGTTATTGCTTGTTGTTTTTTCTTAGGTTTGGGGGTGTTAGTAG	120
H85	GGTTTTGTTGGCTTGTGGTTATTGTTTGTTGTTTTTTCTTAGGTTTGGGGGTGTTAGTAG	120
H84	GGTTTTGTTGGCTTGTGGTTATTGTTTGTTGTTTTTTCTTAGGTTTGGGGGTGTTAGTAG	120
H83	GGTTTTGTTGGCTTGTGGTTATTGTTTGTTGTTTTTTCTTAGGTTTGGGGGTGTTAGTAG	120
H82	GGTTTTGTTGGCTTGTGGTTATTGTTTGTTGTTTTTTCTTAGGTTTGGGGGTGTTAGTAG	120
H81	GGTTTTGTTGGCTTGTGGTTATTGTTTGTTGTTTTTTCTTAGGTTTGGGGGTGTTAGTAG	120
H79	GGTTTTGTTGGCTTGTGGTTATTGTTTGTTGTTTTTTCTTAGGTTTGGAGGTGTTAGTAG	120
H78	GGTTTTGTTGGCTTGTGGTTATTGTTTGTTGTTTTTTCTTAGGTTTGGGGGTGTTAGTAG	120
H77	GGTTTTGTTGGCTTGTGGTTATTGTTTGTTGTTTTTTCTTAGGTTTGGGGGTGTTAGTAG	120
H76	GGTTTTGTTGGCTTGTGGTTATTGTTTGTTGTTTTTTCTTAGGTTTGGGGGTGTTAGTAG	120

```
H75        GGTTTTGTTGGCTTGTGGTTACTGTTTGTTGTTTTTTCTTAGGTTTGGGGGTGTTAGTAG   120
H74        GGCTTTGTTGGCTTGTGGTTATTGTTTGTTGTTTTTTCTTAGGTTTGGGGGTGTTAGTAG   120
H68        GGTTTTGTTGGCTTGTGGTTATTGTTTGTTGTTTTTTCTTAGGTTTGGGGGTGTTAGTAG   120
H67        GGTTTTGTTGGCTTGTGGTTATTGTTTGTTGTTTTTTCTTAGGTTTGGGGGTGTTAGTAG   120
H66        GGTTTTGTTGGCTTGTGGTTATTGTTTGTTGTTTTTTCTTAGGTTTGGGGGTGTTAGTAG   120
H40        GGTTTTGTTGGCTTGTGGCTATTGTTTGTTGTTTTTTCTTAGGTTTGGGGGTGTTAGTAG   120
H38        GGTTTTGTTGGCTTGTGGTTATTGTTTGTTGTTTTTTCTTAGGTTTGGGGGTGTTAGTAG   120
H33        GGTTTTGTTGGCTTGTGGTTATTGTTTGTTGTTTTTTCTCAGGTTTGGGGGTGTTAGTAG   120
H23        GGTTTTGTTGGCTTGTGGTTATTGTTTGTTGTTTTTTCTTAGGTTTGGGGGTGTTAGTAG   120
H17        GGTTTTGTTGGCTTGTGGTTATTGTTTGTTGTTTTTTCTTAGGTTTGGGGGTGTTAGTAG   120
H10        GGTTTTGTTGGCTTGTGGTTATTGTTTGTTGTTTTTTCTTAGGTTTGGGGGTGTTAGTAG   120
H9         GGTTTTGTTGGCTTGTGGTTATTGTTTGTTGTTTTTTCTTAGGTTTGGGGGTGTTAGTAG   120
H2         GGTTTTGTTGGCTTGTGGTTATTGTTTGTTGTTTTTTCTTAGGTTTGGGGGTGTTAGTAG   120
H63        GGTTTTGTTGGCTTGTGGTTATTGTTTGTTGTTTTTTCTTAGGTTTGGGGGTGTTAGTAG   120
H80        GGTTTTGTTGGCTTGCGGTTATTGTTTGTTGTTTTTTCTTAGGTTTGGGGGTGTTAGTAG   120
H65        GGTTTTGTTGGCTTGTGGTTATTGTTTGTTGTTTTTTCTTAGGTTTGGGGGTGTTAGTAG   120
H64        GGTTTTGTTGGCTTGTGGTTATTGTTTGTTGTTTTTTCTTAGGTTTGGGGGTGTTAGTAG   120
H1         GGTTTTGTTGGCTTGTGGTTATTGTTTGTTGTTTTTTCTTAGGTTTGGGGGTGTTAGTAG   120
H62        GGTTTTGTTGGCTTG-GGTTATTGTTTGTTGTTTTTTCTTAGGTTTGGGGGTGTTAGTAG   119
           ** *** **** *** ** ** **  *********** * ***** *   ** **** *

H54        TGTTAAATTTATGTTGTGGTTTTTAGTGGTTACTAGTATGACTGGTTATAGGTTGTTAAG   180
H60        TGTTAAATTTATGTTGTGGTTTTTAGTGGTTACTAGTATGACTGGTTATAGTCTGTTGAG   180
H58        TGTTAAATTTATGTTGTGGTTTTTAGTGGTTACTAGTATGACTGGTTATAGGTTGTTAAG   180
H37        TGTTAAATTTATGTTGTGGTTTTTAGTGGTTACTAGTATGACTGGTTATAGGTTGTTAAG   180
H56        TGTTAAATTTATGTTGTGGTTTTTGGTTGTTACGAGTATGACTGGTTATAGTCTGTTGAG   180
H59        TGTTAAATTTATGTTGTGGTTTTTAGTGGTTACTAGTATGACTGGTTATAGGTTGTTAAG   180
H32        TGTTAAATTTATGTTGTGATTTTTAGTTGTTACGAGTATGACTGGTTATAGTTTGTTGAG   180
H31        TGTTAAATTTATGTTGTGATTTTTAGTTGTTACGAGTATGACTGGTTATAGTTTGTTGAG   180
H55        TGTTAAATTTATGTTGTGGTTTTTAGTGGTTACTAGTATGACTGGTTATAGGTTGTTAAG   180
H51        TGTTAAATTTATGTTGTGGTTTTTGGTTGTTACGAGTATGACTGGTTATAGTCTGTTGAG   180
H35        TGTTAAATTTATGTTGTGGTTTTTAGTTGTTACGAGTATGACTGGCTATAGTTTGTTGAG   180
H36        TGTTAAATTTATGTTGTGGTTTTTAGTGGTTACTAGTATGACTGGTTATAGGTTGTTAAG   180
H34        TGTTAAATTTATGTTGTGATTTTTAGTTGTTACGAGTATGACTGGTTATAGTTTGTTGAG   180
H57        TGTTAAATTTATGTTGTGGTTTTTAGTGGTTACTAGTATGACTGGTTATAGGTTGTTAAG   180
H45        TGTTAAATTTATGTTGTGGTTTTTGGTTGTTACGAGTATGACTGGTTATAGTTTGTTGAG   180
H43        TGTTAAATTTATGTTGTGGTTTTTGGTTGTTACGAGTATGACTGGTTATAGTCTGTTGAG   180
H30        TGTTAAATTTATGTTGTGGTTTTTAGTTGTTACGAGTATGACTGGTTATAGTTTGTTGAG   180
H18        TGTTAAATTTATGTTGTGGTTTTTGGTTGTTACGAGTATTACTGGTTATAGTCTGTTGAG   180
H50        TGTTAAATTTATGTTGTGGTTTTTGGTTGTTACGAGTATGACTGGTTATAGTCTGTTGAG   180
H27        TGTTAAATTTATGTTGTGGTTTTTGGTTGTTACGAGTATGACTGGTTATAGTCTGTTGAG   180
H24        TGTTAAATTTATGTTGTGGTTTTTGGTTGTTACGAGTATGACTGGTTATAGTTTATTGAG   180
H22        TGTTAAATTTATGTTGTGGTTTTTGGTTGTTACGAGTATGACTGGTTATAGTTTATTGAG   180
H15        TGTTAAATTTATGTTGTGGTTTTTGGTTGTTACGAGTATGACTGGTTATAGTTTGTTGAG   180
H5         TGTTAAATTTATGTTGTGGTTTTTGGTTGTTACGAGTATGACTGGTTATAGTTTATTGAG   180
H4         TGTTAAATTTATGTTGTGGTTTTTGGTTGTTACGAGTATGACTGGTTATAGTTTATTGAG   180
H61        TGTTAAATTTATGTTGTGGTTTTTGGTTGTTACGAGTATGACTGGTTATAGTCTGTTGAG   179
H48        TGTTAAATTTATGTTGTGGTTTTTGGTTGTTACGGGTATGACTGGTTATAGTCTGTTGAG   180
H42        TGTTAAATTTATGTTGTGGTTTTTGGTTGTTACGAGTATGACTGGTTATAGTCTGTTGAG   180
H29        TGTTAAATTTATGTTGTGGTTTTTGGTTGTTACGAGTATGACTGGTTATAGTCTGTTGAG   180
H28        TGTTAAATTTATGTTGTGGTTTTTGGTTGTTACGAGTATGACTGGTTATAGTCTGTTGAG   180
H25        TGTTAAATTTATGTTGTGGTTTTTGGTTGTTACGAGTATGACTGGTTATAGTCTGTTGAG   180
H20        TGTTAAATTTATGTTGTGGTTTTTGGTTGTTACGAGTATGGCTGGTTATAGTCTGTTGAG   180
H12        TGTTAAATTTATGTTGTGGTTTTTGGTTGTTACGAGTATGACTGGTTATAGTCTGTTGAG   180
H11        TGTTAAATTTATGTTGTGGTTTTTGGTTGTTACGAGTATGACTGGTTATAGTCTGTTGAG   180
H8         TGTTAAATTTATGTTGTGGTTTTTGGTTGTTACGAGTATGACTGGTTATAGTCTGTTGAG   180
H3         TGTTAAATTTATGTTGTGGTTTTTGGTTGTTACGAGTATGACTGGTTATAGTTTGTTGAG   180
```

```
H73      TGTTAAATTTATGTTGTGGTTTTTGGTTGTTACGAGTATGACTGGTTATAGTCTGTTGAG   180
H72      TGTTAAATTTATGTTGTGGTTTTTGGTTGTTACGAGTATGACTGGTTATAGTCTGTTGAG   180
H71      TGTTAAATTTATGTTGTGGTTTTTGGTTGTTACGAGTATGACTGGTTATAGTCTGTTGAG   180
H70      TGTTAAATTTATGTTGTGGTTTTTGGTTGTTACGAGTATGACTGGTTATAGTCTGTTGAG   180
H69      TGTTAAATTTATGTTGTGGTTTTTGGTTGTTACGAGTATGACTGGTTATAGCCTGTTGAG   180
H53      TGTTAAATTTATGTTGTGGTTTTTGGTTGTTACGAGTATGACTGGTTATAGTCTGTTGAG   180
H52      TGTTAAATTTATGTTGTGGTTTTTGGTTGTTACGAGTATGACTGGTTATAGTCTGTTGAG   180
H49      TGTTAAATTTATGTTGTGGTTTTTGGTTGTTACGGGTATGACTGGTTATAGTCTGTTGAG   180
H47      TGTTAAATTTATGTTGTGGTTTTTGGTTGTTACGAGTATGACTGGTTATAGTCTGTTGAG   180
H46      TGTTAAATTTATGTTGTGGTTTTTGGTTGTTACGAGTATGACTGGTTATAGTTTGTTGAG   180
H44      TGTTAAATTTATGTTGTGGTTTTTGGTTGTTACGAGTATGACTGGTTATAGCCTGTTGAG   180
H41      TGTTAAATTTATGTTGTGGTTTTTGGTTGTTACGAGTATGACTGGTTATAGTCTGTTGAG   180
H39      TGTTAAATTTATGTTGTGGTTTTTGGTTGTTACGAGTATGACTGGTTATAGTCTGTTGAG   180
H26      TGTTAAATTTATGTTGTGGTTTTTGGTTGTTACGAGTATGACTGGTTATAGTCTGTTGAG   180
H21      TGTTAAATTTATGTTGTGGTTTTTGGTTGTTACGAGTATGACTGGTTATAGTCTGTTGAG   180
H19      TGTTAAATTTATGTTGTGGTTTTTGGTTGTTACTAGTATGACTGGTTATAGTCTGTTGAG   180
H16      TGTTAAATTTATGTTGTGGTTTTTGGTTGTTACGAGTATGACTGGTTATAGTCTGTTGAG   180
H14      TGTTAAATTTATGTTGTGGTTTTTGGTTGTTACGAGTATGACTGGTTATAGTCTGTTGAG   180
H13      TGTTAAATTTATGTTGTGGTTTTTGGTTGTTACGAGTATGACTGGTTATAGTCTGTTGAG   180
H7       TGTTAAATTTATGTTGTGGTTTTTGGTTGTTACGAGTATGACTGGTTATAGTCTGTTGAG   180
H6       TGTTAAATTTATGTTGTGGTTTTTGGTTGTTACGAGTATGACTGGTTATAGTCTGTTGAG   180
H91      TGTTAAATTTATGTTGTGGTTTTTGGTTGTTACGAGTATGACTGGTTATAGTCTGTTGAG   180
H90      TGTTAAATTTATGTTGTGGTTTTTGGTTGTTACGAGTATGACTGGTTATAGTCTGTTGAG   180
H89      TGTTAAATTTATGTTGTGGTTTTTGGTTGTTACGAGTGTGACTGGTTATAGTCTGTTGAG   180
H88      TGTTAAATTTATGTTGTGGTTTTTGGTTGTTACGAGTATGACTGGTTATAGTCTGTTGAG   180
H87      TGTTAAATTTATGTTGTGGTTTTTGGTTGTTACGAGTATGACTGGTTATAGTCTGTTGAG   180
H86      TGTTAAATTTATGTTGTGGTTTTTGGTTGTTACGAGTATGACTGGTTATAGTCTGTTGAG   180
H85      TGTTAAATTTATGTTGTGGTTTTTGGTTGTTACGAGTATGACTGGTTATAGCCTGTTGAG   180
H84      TGTTAAATTTATGTTGTGGTTTTTGGTTGTTACGAGTATGACTGGTTATAGTCTGTTGAG   180
H83      TGTTAAATTTATGTTGTGGTTTTTGGTTGTTACGAGTATGACCGGTTATAGTCTGTTGAG   180
H82      TGTTAAATTTATGTTGTGGTTTTTGGTTGTTACGAGTATGACTGGTTATAGTCTGTTGAG   180
H81      TGTTAAATTTATGTTGTGGTTTTTGGTTGTTACGAGTATGACTGGTTATAGTCTGTTGAG   180
H79      TGTTAAATTTATGTTGTGGTTTTTGGTTGTTACGAGTATGACTGGTTATAGTCTGTTGAG   180
H78      TGTTAAATTTATGTTGTGGTTTTTGGTTGTTACGAGTATGACTGGTTATAGTCTGTTGAG   180
H77      TGTTAAATTTATGTTGTGGTTTTTGGTTGTTACGAGTATGACTGGTTATAGTCTGTTGAG   180
H76      TGTTAAATTTATGTTGTGGTTTTTGGTTGTTGCGAGTATGACTGGTTATAGTCTGTTGAG   180
H75      TGTTAAATTTATGTTGTGGTTTTTGGTTGTTACGAGTATGACTGGTTATAGTCTGTTGAG   180
H74      TGTTAAATTTATGTTGTGGTTTTTGGTTGTTACGAGTATGACTGGTTATAGTCTGTTGAG   180
H68      TGTTAAATTTATGTTGTGGTTTTTGGTTGTTACGAGTATGACTGGCTATAGTCTGTTGAG   180
H67      TGTTAAATTTATGTTGTGGTTTTTGGTTGTTACGAGTATGACTGGTTATAGTCTGTTAAG   180
H66      TGTTAAATTTATGTTGTGGTTTTTGGTTGTTACGAGTATGACTGGTTATAGTCTGTTGAG   180
H40      TGTTAAATTTATGTTGTGGTTTTTGGTTGTTACGAGTATGACTGGTTATAGTCTGTTGAG   180
H38      TGTTAAATTTATGTTGTGGTTTTTGGTTGTTACGAGTATGACTGGTTATAGTCTGTTGAG   180
H33      TGTTAAATTTATGTTGTGGTTTTTGGTTGTTACGAGTATGACTGGTTATAGTCTGTTGAG   180
H23      TGTTAAATTTATGTTGTGGTTTTTGGTTGTTACGAGTATGACTGGTTATAGTCTGTTGAG   180
H17      TGTTAAATTTATGTTGTGGTTTTTGGTTGTTACGAGTATGACTGGTTATAGTCTGTTGAG   180
H10      TGTTAAATTTATGTTGTGGTTTTTGGTTGTTACGAGTATGACTGGTTATAGTCTGTTGAG   180
H9       TGTTAAATTTATGTTGTGGTTTTTGGTTGTTACGAGTATGACTGGTTATAGTTTGTTGAG   180
H2       TGTTAAATTTATGTTGTGGTTTTTGGTTGTTACGAGTATGACTGGTTATAGTCTGTTGAG   180
H63      TGTTAAATTTATGTTGTGGTTTT-TGTTGTTACGAGTATGACTGGTTATAGTCTGTTGAG   179
H80      TGTTAAATTTATGTTGTGGTTTTTGGTTGTTACGAGTATGACTGGTTATAGTCTGTTGAG   180
H65      TGTTAAATTTATGTTGTGGTTT-TTGTTGTTACGAGTATGACTGGTTATAGTCTGTTGAG   179
H64      TGTTAAATTTATGTTGTGGTTTTTGGTTGTTACGAGTATGACTGGTTATAGTCTGTTGAG   180
H1       TGTTAAATTTATGTTGTGGTTTTTGGTTGTTACGAGTATGACTGGTTATAGTCTGTTGAG   180
H62      TGTTAAATTTATGTTGTGGTTTTTGGTTGTTACGAGTATGACTGGTTATAGTCTGTTGAG   179
         ****************** ***   ** *** *  ** *  * ** *****  * ** **
```

H54	TGTTGGTTGGGGTTGTTATAATAAGTTTGCTTTGGTTAGCTGTGTTCGTTCTGCTTTTGG	240
H60	TGTTGGTTGGGGTTGTTATAATAAGTTTGCTTTGGTTAGCTGTGTTCGTTCTGCTTTTGG	240
H58	TGTTGGTTGGGGTTGTTATAATAAGTTTGCTTTGGTTAGCTGTGTTCGTTCTGCTTTTGG	240
H37	*TGTTGGTTGGGGTTGTTATAATAAGTTTGCTTTGGTTAGCTGTGTTCGTTCTGCTTTTGG*	*240*
H56	TGTTGGTTGGGGTTGTTATAATAAGTTTGCTTTGGTTAGCTGTGTTCGTTCTGCTTTTGG	240
H59	TGTTGGTTGGGGTTGTTATAATAAGTTTGCTTTGGTTAGCTGTGTTCGTTCTGCTTTTGG	240
H32	TGTTGGTTGGGGTTGTTATAATAAGTTTGCTTTGGTGAGCTGTGTTCGTTCTGCTTTTGG	240
H31	TGTTGGTTGGGGTTGTTATAATAAGTTTGCTTTGGTGAGCTGTGTTCGTTCTGCTTTTGG	240
H55	TGTTGGTTGGGGTTGTTATAATAAGTTTGCTTTGGTTAGCTGTGTTCGTTCTGCTTTTGG	240
H51	TGTTGGTTGGGGTTGTTATAATAAGTTTGCTTTGGTGAGTTGTGTTCGTTCTGCTTTTGG	240
H35	TGTTGGTTGGGGTTGTTATAATAAGTTTGCTTTGGTAAGTTGTGTTCGTTCTGCTTTTGG	240
H36	*TGTTGGTTGGGGTTGTTATAATAAGTTTGCTTTGGTTAGCTGTGTTCGTTCTGCTTTTGG*	*240*
H34	TGTTGGTTGGGGTTGTTATAATAAGTTTGCTTTGGTGAGTTGTGTTCGTTCTGCTTTTGG	240
H57	TGTTGGTTGGGGTTGTTATAATAAGTTTGCTTTGGTGAGTTGTGTTCGTTCTGCTTTTGG	240
H45	TGTTGGTTGGGGTTGTTATAATAAGTTTGCTTTGGTGAGTTGTGTTCGTTCTGCTTTTGG	240
H43	TGTTGGTTGGGGTTGTTATAATAAGTTTGCTTTGGTGAGTTGTGTTCGTTCTGCTTTTGG	240
H30	TGTTGGTTGGGGTTGTTATAATAAGTTTGCTTTGGTGAGTTGTGTTCGTTCTGCTTTTGG	240
H18	TGTTGGTTGGGGTTGTTATAATAAGTTTGCTTTGGTGAGTTGTGTTCGTTCTGCTTTTGG	240
H50	TGTTGGTTGGGGTTGTTATAATAAGTTTGCTTTGGTGAGTTGTGTTCGTTCTGCTTTTGG	240
H27	TGTTGGTTGGGGTTGTTATAATAAGTTTGCTTTGGTGAGTTGTGTTCGTTCTGCTTTTGG	240
H24	TGTTGGTTGGGGTTGTTATAATAAGTTTGCTTTGGTGAGTTGTGTTCGTTCTGCTTTTGG	240
H22	TGTTGGTTGGGGTTGTTATAATAAGTTTGCTTTGGTGAGTTGTGTTCGTTCTGCTTTTGG	240
H15	TGTTGGTTGGGGTTGTTATAATAAGTTTGCTTTGGTGAGTTGTGTTCGTTCTGCTTTTGG	240
H5	TGTTGGTTGGGGTTGTTATAATAAGTTTGCTTTGGTGAGTTGTGTTCGTTCTGCTTTTGG	240
H4	TGTTGGTTGGGGTTGTTATAATAAGTTTGCTTTGGTGAGTTGTGTTCGTTCTGCTTTTGG	240
H61	TGTTGGTTGGGGTTGTTATAATAAGTTTGCTTTGGTGAGTTGTGTTCGTTCTGCTTTTGG	239
H48	TGTTGGTTGGGGTTGTTATAATAAGTTTGCTTTGGTGAGTTGTGTTCGTTCTGCTTTTGG	240
H42	TGTTGGTTGGGGGAGTTATAATAAGTTTGCTTTGGTGAGTTGTGTTCGTTCTGCTTTTGG	240
H29	TGTTGGTTGGGGTTGTTATAATAAGTTTGCTTTGGTGAGTTGTGTTCGTTCTGCTTTTGG	240
H28	TGTTGGTTGGGGTTGTTATAATAAGTTTGCTTTGGTGAGTTGTGTTCGTTCTGCTTTTGG	240
H25	TGTTGGCTGGGGTTGTTATAATAAGTTTGCTTTGGTGAGTTGTGTTCGTTCTGCTTTTGC	240
H20	TGTTGGTTGGGGTTGTTATAATAAGTTTGCTTTGGTGAGTTGTGTTCGTTCTGCTTTTGG	240
H12	TGTTGGTTGGGGTTGTTATAATAAGTTTGCTTTGGTGAGTTGTGTTCGTTCTGCTTTTGG	240
H11	TGTTGGTTGGGGTTGTTATAATAAGTTTGATTTGGTGAGTTGTGTTCGTTCTGCTTTTGG	240
H8	TGTTGGTTGGGGTTGTTATAATAAGTTTGCTTTGGTGAGTTGTGTTCGTTCTGCTTTTGG	240
H3	TGTTGGTTGGGGTTGTTATAATAAGTTTGCTTTGGTGAGTTGTGTTCGTTCTGCTTTTGG	240
H73	TGTTGGTTGGGGTTGTTATAATAAGTTTGCTTTGGTGAGTTGTGTTCGTTCTGCTTTTGG	240
H72	TGTTGGTTGGGGTTGTTATAATAAGTTTGCTTTGGTGAGTTGTGTTCGTTCTGCTTTTGG	240
H71	TGTTGGTTGGGGTTGTTATAATAAGTTTGCTTTGGTGAGTTGTGTTCGTTCTGCTTTTGG	240
H70	TGTTGGTTGGGGTTGTTATAATAAGTTTGCTTTGGTGAGTTGTGTTCGTTCTGCTTTTGG	240
H69	TGTTGGTTGGGGTTGTTATAATAAGTTTGCTTTGGTGAGTTGTGTTCGTTCTGCTTTTGG	240
H53	TGTTGGTTGGGGTTGTTATAATAAGTTTGCTTTGGTGAGTTGTGTTCGTTCTGCTTTTGG	240
H52	TGTTGGTTGGGGTTGTTATAATAAGTTTGCTTTGGTGAGTTGTGTTCGTTCTGCTTTTGG	240
H49	TGTTGGTTGGGGTTGTTATAATAAGTTTGCTTTGGTGAGTTGTGTTCGTTCTGCTTTTGG	240
H47	TGTTGGCTGGGGTTGTTATAATAAGTTTGCTTTGGTGAGTTGTGTTCGTTCTGCTTTTGG	240
H46	TGTTGGTTGGGGTTGTTATAATAAGTTTGCTTTGGTGAGTTGTGTTCGTTCTGCTTTTGG	240
H44	TGTTGGTTGGGGTTGTTATAATAAGTTTGCTTTGGTGAGTTGTGTTCGTTCTGCTTTTGG	240
H41	TGTTGGTTGGGGTTGTTATAATAAGTTTGCTTTGGTGAGTTGTGTTCGTTCTGCTTTTGG	240
H39	TGTTGGTTGGGGGAGTTATAATAAGTTTGCTTTGGTGAGTTGTGTTCGTTCTGCTTTTGG	240
H26	TGTTGGTTGGGGTTGTTATAATAAGTTTGCTTTAGTGAGTTGTGTTCGTTCTGCTTTTGG	240
H21	TGTTGGCTGGGGTTGTTATAATAAGTTTGATTTGGTGAGTTGTGTTCGTTCTGCTTTTGG	240
H19	TGTTGGCTGGGGTTGTTATAATAAGTTTGCTTTGGTGAGTTGTGTTCGTTCTGCTTTTGG	240
H16	TGTTGGTTGGGGTTGTTATAATAAGTTTGCTTTGGTGAGTTGTGTTCGTTCTGCTTTTGG	240
H14	TGTTGGTTGGGGTTGTTATAATAAGTTTGCTTTGGTGAGTTGTGTTCGTTCTGCTTTTGG	240
H13	TGTTGGTTGGGGTTGTTATAATAAGTTTGCTTTGGTGAGTTGTGTTCGTTCTGCTTTTGG	240
H7	TGTTGGTTGGGGTTGTTATAATAAGTTTGCTTTGGTGAGTTGTGTTCGTTCTGCTTTTGG	240
H6	TGTTGGTTGGGGTTGTTATAATAAGTTTGCTTTGGTGAGTTGTGTTCGTTCTGATTTTGG	240

```
H91      TGTTGGTTGGGGTTGTTATAATAAGTTTGCTTTGGTGAGTTGTGTTCGTTCTGCTTTTGG   240
H90      TGTTGGTTGGGGTTGTTATAATAAGTTTGCTTTGGTGAGTTGTGTTCGTTCTGCTTTTGG   240
H89      TGTTGGTTGGGGTTGTTATAATAAGTTTGCTTTGGTGAGTTGTGTTCGTTCTGCTTTTGG   240
H88      TGTTGGTTGGGGTTGTTATAATAAGTTTGCTTTGGTGAGTTGTGTTCGTTCTGCTTTTGG   240
H87      TGTTGGTTGGGGTTGTTATAATAAGTTTGCTTTGGTGAGTTGTGTTCGTTCTGCTTTTGG   240
H86      TGTTGGTTGGGGTTGTTATAATAAGTTTGCTTTGGTGAGTTGTGTTCGTTCTGCTTTTGG   240
H85      TGTTGGTTGGGGTTGTTATAATAAGTTTGCTTTGGTGAGTTGTGTTCGTTCTGCTTTTGG   240
H84      TGTTGGTTGGGGTTGTTATAATAAGTTCGCTTTGGTGAGTTGTGTTCGTTCTGCTTTTGG   240
H83      TGTTGGTTGGGGTTGTTATAATAAGTTTGCTTTGGTGAGTTGTGTTCGTTCTGCTTTTGG   240
H82      TGTTGGTTGGGGTTGTTATAATAAGTTTGCTTTGGTGAGTTGTGTTCGTTCTGCTTTTGG   240
H81      TGTTGGTTGGGGTTGTTATAATAAGTTTGCTTTGGTGAGTTGTGTTCGTTCTGCTTTTGG   240
H79      TGTTGGTTGGGGTTGTTATAATAAGTTTGCTTTGGTGAGTTGTGTTCGTTCTGCTTTTGG   240
H78      TGTTGGTTGGGGTTGTTATAATAAGTTTGCTTTGGTGAGTTGTGTTCGTTCTGCTTTTGG   240
H77      TGTTGGTTGGGGTTGTTATAATAAGTTTGCTTTGGTGAGTTGTGTTCGTTCTGCTTTTGG   240
H76      TGTTGGTTGGGGTTGTTATAATAAGTTTGCTTTGGTGAGTTGTGTTCGTTCTGCTTTTGG   240
H75      TGTTGGTTGGGGTTGTTATAATAAGTTTGCTTTGGTGAGTTGTGTTCGTTCTGCTTTTGG   240
H74      TGTTGGTTGGGGTTGTTATAATAAGTTTGCTTTGGTGAGTTGTGTTCGTTCTGCTTTTGG   240
H68      TGTTGGTTGGGGTTGTTATAATAAGTTTGCTTTGGTGAGTTGTGTTCGTTCTGCTTTTGG   240
H67      TGTTGGTTGGGGTTGTTATAATAAGTTTGCTTTGGTGAGTTGTGTTCGTTCTGCTTTTGG   240
H66      TGTTGGTTGGGGTTGTTATAATAAGTTTGCTTTGGTGAGTTGTGTTCGTTCTGCTTTTGG   240
H40      TGTTGGTTGGGGTTGTTATAATAAGTTTGCTTTGGTGAGTTGTGTTCGTTCTGCTTTTGG   240
H38      TGTTGGTTGGGGTTGTTATAATAAGTTTGCTTTGGTGAGTTGTGTTCGTTCTGCTTTTGG   240
H33      TGTTGGTTGGGGTTGTTATAATAAGTTTGCTTTGGTGAGTTGTGTTCGTTCTGCTTTTGG   240
H23      TGTTGGTTGGGGTTGTTATAATAAGTTTGCTTTGGTGAGTTGTGTTCGTTCTGCTTTTGG   240
H17      TGTTGGCTGGGGTTGTTATAATAAGTTTGCTTTGGTGAGTTGTGTTCGTTCTGCTTTTGG   240
H10      TGTTGGTTGGGGTTGTTATAATAAGTTTGCTTTAGTGAGTTGTGTTCGTTCTGCTTTTGG   240
H9       TGTTGGTTGGGGTTGTTATAATAAGTTTGCTTTGGTGAGTTGTGTTCGTTCTGCTTTTGG   240
H2       TGTTGGTTGGGGTTGTTATAATAAGTTTGCTTTGGTGAGTTGTGTTCGTTCTGCTTTTGG   240
H63      TGTTGGTTGGGGTTGTTATAATA-GTTTGCTTTGGTGAGTTGTGTTCGTTCTGCTTTTGG   238
H80      TGTTGGTTGGGGTTGTTATAATAAGTTTGCTTTGGTGAGTTGTGTTCGTTCTGCTTTTGG   240
H65      TGTTGGTTGGGGTTGTTATAATAAGTTTGCTTTGGTGAGTTGTGTTCGTTCTGCTTTTGG   239
H64      TGTTGGTTGGGGTTGTTATAATA-GTTTGCTTTGGTGAGTTGTGTTCGTTCTGCTTTTGG   239
H1       TGTTGGTTGGGGTTGTTATAATAAGTTTGCTTTGGTGAGTTGTGTTCGTTCTGCTTTTGG   240
H62      TGTTGGTTGGGGTTGTTATAATAAGTTTGCTTTGGTGAGTTGTGTTCGTTCTGCTTTTGG   239
         ****** *****   ********* *** * *** ** ** ************* *****

H54      GTCTGTTAGGTTTGAGGCTTGTTTTATGTGTATTGTTGTTTTGGTTGCATTGGTTTGGGG   300
H60      GTCTGTTAGGTTTGAGGCTTGTTTTATGTGTATTGTTGTTTTGGTTGCATTGGTTTGGGG   300
H58      GTCGGTTAGTTTTGAGGCTTGTTTTATGTGTATTGTTGTTTTAGTTGCATTGGTTTGGGG   300
H37      GTCTGTTAGGTTTGAGGCTTGTTTTATGTGTATTGTTGTTTTGGTTGCATTGGTTTGGGG   300
H56      GTCTGTTAGGTTTGAGGCTTGTTTTATGTGTATTGTTGTTTTGGTTGCATTGGTTTGGGG   300
H59      GTCTGTTAGGTTTGAGGCTTGTTTTATGTGTATTGTTGTTTTAGTTGCTTTGGTTTGGGG   300
H32      GTCTGTTAGTTTTGAGGCTTGTTTTATGTGTATTGTTGTTTTGGTTGCTTTGGTTTGGGG   300
H31      GTCTGTTAGTTTTGAGGCTTGTTTTATGTGTATTGTTGTTTTAGTTGCTTTGGTTTGGGG   300
H55      GTCTGTTAGGTTTGAGGCTTGTTTTATGTGTATTGTTGTTTTAGTTGCTTTGGTTTGGGG   300
H51      GTCGGTTAGTTTTGAGGCTTGTTTTATGTGTATTGTTGTTTTGGTTGCATTGGTTTGGGG   300
H35      GTCGGTTAGTTTTGAGGCTTGTTTTATGTGTATTGTCGTTTTAGTTGCTTTGGTTTGGGG   300
H36      GTCTGTTAGGTTTGAGGCTTGTTTTATGTGTATTGTTGTTTTGGTTGCATTGGTTTGGGG   300
H34      GTCGGTTAGTTTTGAGGCTTGTTTTATGTGTATTGTTGTTTTAGTTGCTTTGGTTTGGGG   300
H57      GTCGGTTAGTTTTGAGGCTTGTTTTATGTGTATTGTTGTTTTAGTTGCTTTGGTTTGGGG   300
H45      GTCGGTTAGTTTTGAGGCTTGTTTTATGTGTATTGTTGTTTTAGTTGCTTTGGTTTGGGG   300
H43      GTCGGTTAGTTTTGAGGCTTGTTTTATGTGTATTGTTGTTTTGGTTGCATTGGTTTGGGG   300
H30      GTCGGTTAGTTTTGAGGCTTGTTTTATGTGTATTGTCGTTTTAGTTGCTTTGGTTTGGGG   300
H18      GTCGGTTAGTTTTGAGGCTTGTTTTATGTGTATTGTTGTTTTAGTTGCTTTGGTTTGGGG   300
H50      GTCGGTTAGTTTTGAGGCTTGTTTTATGTGTATTGTTGTTTTAGTTGCTTTGGTTTGGGG   300
H27      GTCGGTTAGTTTTGAGGATTGTTTTATGTGTATCGTTGTTTTAGTTGCTTTGGTTTGGGG   300
H24      GTCGGTTAGTTTTGAGGCTTGTTTTATGTGTATTGTTGTTTTAGTTGCTTTGGTTTGGGG   300
```

H22	GTCGGTTAGTTTTGAGGCTTGTTTTATGTGTATTGTCGTTTTAGTTGATTTGGTTTGGGG	300
H15	GTCGGTTAGTTTTGAGGCTTGTTTTATGTGTATTGTTGTTTTAGTTGCTTTGGTTTGGGG	300
H5	GTCGGTTAGTTTTGAGGCTTGTTTTATGTGTATTGTCGTTTTAGTTGCTTTGGTTTGGGG	300
H4	GTCGGTTAGTTTTGAGGATTGTTTTATGTGTATTGTCGTTTTAGTTGCTTTGGTTTGGGG	300
H61	GTCGGTTAGTTTTGAGGCTTGTTTTATGTGTATTGTCGTTTTAGTTGCTTTGGTTTGGGG	299
H48	GTCGGTTAGTTTTGAGGCTTGTTTTATGTGTATTGTTGTTTTAGTTGCTTTGGTTTGGGG	300
H42	GTCGGTTAGTTTTGAGGCTTGTTTTATGTGTATTGTTGTTTTAGTTGCTTTGGTTTGGGG	300
H29	GTCGGTTAGTTTTGAGGCTTGTTTTATGTGTATTGTTGTTTTAGTTGATTTGGTTTGGGG	300
H28	GTCGGTTAGTTTTGAGGCTTGTTTTATGTGTATTGTTGTTTTAGTTGCTTTGGTTTGGGG	300
H25	GTCGGTTAGTTTTGAGGCTTGTTTTATGTGTATTGTCGTTTTAGTTGCTTTGGTTTGGGG	300
H20	GTCGGTTAGTTTTGAGGCTTGTTTTATGTGTATTGTTGTTTTAGTTGCTTTGGTTTGGGG	300
H12	GTCGGTTAGTTTTGAGGATTGTTTTATGTGTATTGCTGTTTTAGTTGCTTTGGTTTGGGG	300
H11	GTCGGTTAGTTTTGAGGCTTGTTTTATGTGTATTGCTGTTTTAGTTGCTTTGGTTTGGGG	300
H8	GTCGGTTAGTTTTGAGGCTTGTTTTATGTGTATTGTTGTTTTAGTTGCTTTGGTTTGGGG	300
H3	GTCGGTTAGTTTTGAGGCTTGTTTTATGTGTATTGTTGTTTTAGTTGCTCTGGTTTGGGG	300
H73	GTCGGTTAGTTTTGAGGCTTGTTTTATGTGTGTTGTCGTTTTAGTTGCTTTGGTTTGGGG	300
H72	GTCGGTTAGTTTTGAGGCTTGTTTTATGTGTGTTGTCGTTTTAGTTGCTTTGGTTTGGGG	300
H71	GTCGGTTAGTTTTGAGGCTTGTTTTATGTGTGTTGTCGTTTTAGTTGCTTTGGTTTGGGG	300
H70	GTCGGTTAGTTTTGAGGCTTGTTTTATGTGTGTTGTCGTTTTAGTTGCTTTGGTTTGGGG	300
H69	GTCGGTTAGTTTTGAGGCTTGTTTTATGTGTGTTGTCGTTTTAGTTGCTTTGGTTTGGGG	300
H53	GTCGGTTAGTTTTGAGGCTTGTTTTATGTGTATTGTTGTTTTAGTTGCTTTGGTTTGGGG	300
H52	GTCGGTTAGTTTTGAGGCTTGTTTTATGTGTATTGTTGTTTTAGTTGCTTTGGTTTGGGG	300
H49	GTCGGTTAGTTTTGAGGCTTGTTTTATGTGTATTGTTGTTTTAGTTGCTTTGGTTTGGGG	300
H47	GTCGGTTAGTTTTGAGGCTTGTTTTATGTGTATTGTTGTTTTAGTTGCTTTGGTTTGGGG	300
H46	GTCGGTTAGTTTTGAGGCTTGTTTTATGTGTATTGTTGTTTTAGTTGCTTTGGTTTGGGG	300
H44	GTCGGTTAGTTTTGAGGCTTGTTTTATGTGTATTGTTGTTTTAGTTGCTTTGGTTTGGGG	300
H41	GTCGGTTAGTTTTGAGGCTTGTTTTATGTGTATTGTTGTTTTAGTTGCTTTGGTTTGGGG	300
H39	GTCGGTTAGTTTTGAGGCTTGTTTTATGTGTATTGTCGTTTTAGTTGCTTTGGTTTGGGG	300
H26	GTCGGTTAGTTTTGAGGCTTGTTTTATGTGTATTGTCGTTTTAGTTGCTTTGGTTTGGGG	300
H21	GTCGGTTAGTTTTGAGGCTTGTTTTATGTGTATTGTCGTTTTAGTTGCTTTGGTTTGGGG	300
H19	GTCGGTTAGTTTTGAGGCTTGTTTTATGTGTATTGTCGTTTTAGTTGCTTTGGTTTGGGG	300
H16	GTCGGTTAGTTTTGAGGCTTGTTTTATGTGTATTGTTGTTTTAGTTGCTTTGGTTTGGGG	300
H14	GTCGGTTAGTTTTGAGGATTGTTTTATGTGTATTGTTGTTTTAGTTGCTTTGGTTTGGGG	300
H13	GTCGGTTAGTTTTGAGGCTTGTTTTATGTGTATTGTTGTTTTAGTTGATTTGGTTTGGGG	300
H7	GTCGGTTAGTTTTGAGGATTGTTTTATGTGTATTGTCGTTTTAGTTGCTTTGGTTTGGGG	300
H6	GTCGGTTAGTTTTGAGGCTTGTTTTATGTGTATTGTCGTTTTAGTTGCTTTGGTTTGGGG	300
H91	GTCGGTTAGTTTTGAGGCTTGTTTTATGTGTATTGTCGTTTTAGTTGCTTTGGTTTGGGG	300
H90	GTCGGTTAGTTTTGAGGCTTGTTTTATGTGTATTGTCGTTTTAGTTGCTTTGGTTTGGGG	300
H89	GTCGGTTAGTTTTGAGGCTTGTTTTATGTGTATTGTCGTTTTAGTTGCTTTGGTTTGGGG	300
H88	GTCGGTTAGTTTTGAGGCTTGTTTTATGTGTATTGTCGTTTTAGTTGCTTTGGTTTGGGG	300
H87	GTCGGTTAGTTTTGAGGCTTGTTTTATGTGTATTGTCGTTTTAGTTGCTTTGGTTTGGGG	300
H86	GTCGGTTAGTTTTGAGGCTTGTTTTATGTGTATTGTCGTTTTAGTTGCTTTGGTTTGGGG	300
H85	GTCGGTTAGTTTTGAGGCTTGTTTTATGTGTATTGTCGTTTTAGTTGCTTTGGTTTGGGG	300
H84	GTCGGTTAGTTTTGAGGCTTGTTTTATGTGTATTGTCGTTTTAGTTGCTTTGGTTTGGGG	300
H83	GTCGGTTAGTTTTGAGGCTTGTTTTATGTGTATTGTCGTTTTAGTTGCTTTGGTTTGGGG	300
H82	GTCGGTTAGTTTTGAGGCTTGTTTTATGTGTATTGTCGTTTTAGTTGCTTTGGTTTGGGG	300
H81	GTCGGTTAGTTTTGAGGCTTGTTTTATGTGTATTGTCGTTTTAGTTGCCTTGGTTTGGGG	300
H79	GTCGGTTAGTTTTGAGGCTTGTTTTATGTGTATTGTCGTTTTAGTTGCTTTGGTTTGGGG	300
H78	GTCGGTTAGTTTTGAGGCTTGTTTTATGTGTATTGTCGTTTTAGTTGCTTTGGTTTGGGG	300
H77	GTCGGTTAGTTTTGAGGCTTGTTTTATGTGTATTGTCGTTTTAGTTGCTTTAGTTTGGGG	300
H76	GTCGGTTAGTTTTGAGGCTTGTTTTATGTGTATTGTCGTTTTAGTTGCTTTGGTTTGGGG	300
H75	GTCGGTTAGTTTTGAGGCTTGTTTTATGTGTATTGTCGTTTTAGTTGCTTTGGTTTGGGG	300
H74	GTCGGTTAGTTTTGAGGCTTGTTTTATGTGTATTGTCGTTTTAGTTGCTTTGGTTTGGGG	300
H68	GTCGGTTAGTTTTGAGGCTTGTTTTATGTGTATTGTCGTTTTAGTTGCTTTGGTTTGGGG	300
H67	GTCGGTTAGTTTTGAGGCTTGTTTTATGTGTATTGTCGTTTTAGTTGCTTTGGTTTGGGG	300
H66	GTCGGTTAGTTTTGAGGCTTGTTTTATGTGTATTGTCGTTTTAGTTGCTTTGGTTTGGGG	300
H40	GTCGGTTAGTTTTGAGGCTTGTTTTATGTGTATTGTCGTTTTAGTTGCTTTGGTTTGGGG	300

```
H38      GTCGGTTAGTTTTGAGGCTTGTTTTATGTGTGTTGTCGTTTTAGTTGCTTTGGTTTGGGG   300
H33      GTCGGTTAGTTTTGAGGCTTGTTTTATGTGTATTGTCGTTTTAGTTGCTTTGGTTTGGGG   300
H23      GTCGGTTAGTTTTGAGGCTTGTTTTATGTGTATTGTCGTTTTAGTTGCTTTGGTTTGGGG   300
H17      GTCGGTTAGTTTTGAGGCTTGTTTTATGTGTATTGTCGTTTTAGTTGCTTTGGTTTGGGG   300
H10      GTCGGTTAGTTTTGAGGCTTGTTTTATGTGTATTGTCGTTTTAGTTGCTTTGGTTTGGGG   300
H9       GTCGGTTAGTTTTGAGGCTTGTTTTATGTGTATTGTCGTTTTAGTTGCTTTGGTTTGGGG   300
H2       GTCGGTTAGTTTTGAGGCTTGTTTTATGTGTATTGTTGTTTTAGTTGCTTTGGTTTGGGG   300
H63      GTCGGTTAGTTTTGAGGCTTGTTTTATGTGTATTGTCGTTTTAGTTGCTTTGGTTTGGGG   298
H80      GTCGGTTAGTTTTGAGGCTTGTTTTATGTGTATTGTCGTTTTAGTTGCTTTGGTTTGGGG   300
H65      GTCGGTTAGTTTTGAGGCTTGTTTTATGTGTATTGTCGTTTTAGTTGCTTTGGTTTGGGG   299
H64      GTCGGTTAGTTTTGAGGCTTGTTTTATGTGTATTGTCGTTTTAGTTGCTTTGGTTTGGGG   299
H1       GTCGGTTAGTTTTGAGGCTTGTTTTATGTGTATTGTCGTTTTAGTTGCTTTGGTTTGGGG   300
H62      GTCGGTTAGTTTTGAGGCTTGTTTTATGTGTATTGTCGTTTTAGTTGCTTTGGTTTGGGG   299
         *** ***** ******* ************* * *  ***** ****   * ********

H54      GAGTTATGG      309
H60      GAGTTATGG      309
H58      GAGTTATGG      309
H37      GAGTTATGG      309
H56      GAGTTATGG      309
H59      GAGTTATGG      309
H32      GAGTTATGG      309
H31      GAGTTATGG      309
H55      GAGTTATGG      309
H51      GAGTTATGG      309
H35      GAGTTATGG      309
H36      GAGTTATGG      309
H34      GAGTTATGG      309
H57      GAGTTATGG      309
H45      GAGTTATGG      309
H43      GAGTTATGG      309
H30      GAGTTATGG      309
H18      GAGTTATGG      309
H50      GAGTTATGG      309
H27      GAGTTATGG      309
H24      GAGTTATGG      309
H22      GAGTTATGG      309
H15      GAGTTATGG      309
H5       GAGTTATGG      309
H4       GAGTTATGG      309
H61      GAGTTATGG      308
H48      GAGTTATGG      309
H42      GAGTTATGG      309
H29      GAGTTATGG      309
H28      GAGTTATGG      309
H25      GAGTTATGG      309
H20      GAGTTATGG      309
H12      GAGTTATGG      309
H11      GAGTTATGG      309
H8       GAGTTATGG      309
H3       GAGTTATGG      309
H73      GAGTTATGG      309
H72      GAGTTATGG      309
H71      GAGTTATGG      309
H70      GAGTTATGG      309
H69      GAGTTATGG      309
H53      GAGTTATGG      309
```

```
H52       GAGTTATGG      309
H49       GAGTTATGG      309
H47       GAGTTATGG      309
H46       GAGTTATGG      309
H44       GAGTTATGG      309
H41       GAGTTATGG      309
H39       GAGTTATGG      309
H26       GAGTTATGG      309
H21       GAGTTATGG      309
H19       GAGTTATGG      309
H16       GAGTTATGG      309
H14       GAGTTATGG      309
H13       GAGTTATGG      309
H7        GAGTTATGG      309
H6        GAGTTATGG      309
H91       GAGTTATGG      309
H90       GAGTTATGG      309
H89       GAGTTATGG      309
H88       GGGTTATGG      309
H87       GAGTTATGG      309
H86       GAGTTATGG      309
H85       GAGTTATGG      309
H84       GAGTTATGG      309
H83       GAGTTATGG      309
H82       GAGTTATGG      309
H81       GAGTTATGG      309
H79       GAGTTATGG      309
H78       AAGTTATGG      309
H77       GAGTTATGG      309
H76       GAGTTATGG      309
H75       GAGTTATGG      309
H74       GAGTTATGG      309
H68       GAGTTATGG      309
H67       GAGTTATGG      309
H66       GAGTTATGG      309
H40       GAGTTATGG      309
H38       GAGTTATGG      309
H33       GAGTTATGG      309
H23       GAGTTATGG      309
H17       GAGTTATGG      309
H10       GAGTTATGG      309
H9        GAGTTATGG      309
H2        GAGTTATGG      309
H63       GAGTTATGG      307
H80       GAGTTATGG      309
H65       GAGTTATGG      308
H64       GAGTTATGG      308
H1        GAGTTATGG      309
H62       GAGTTATGG      308
            *******
```
